# Calmodulin-Mediated Regulation of Gap Junction Channels

**DOI:** 10.3390/ijms21020485

**Published:** 2020-01-12

**Authors:** Camillo Peracchia

**Affiliations:** Department of Pharmacology and Physiology, School of Medicine and Dentistry, University of Rochester, Rochester, NY 14642, USA; camillo.peracchia@gmail.com

**Keywords:** gap junctions, connexins, innexins, calmodulin, membrane channels, channel gating, calcium, pH, chemical gating, voltage gating

## Abstract

Evidence that neighboring cells uncouple from each other as one dies surfaced in the late 19th century, but it took almost a century for scientists to start understanding the uncoupling mechanism (chemical gating). The role of cytosolic free calcium (Ca^2+^_i_) in cell–cell channel gating was first reported in the mid-sixties. In these studies, only micromolar [Ca^2+^]_i_ were believed to affect gating—concentrations reachable only in cell death, which would discard Ca^2+^_i_ as a fine modulator of cell coupling. More recently, however, numerous researchers, including us, have reported the effectiveness of nanomolar [Ca^2+^]_i_. Since connexins do not have high-affinity calcium sites, the effectiveness of nanomolar [Ca^2+^]_i_ suggests the role of Ca-modulated proteins, with calmodulin (CaM) being most obvious. Indeed, in 1981 we first reported that a CaM-inhibitor prevents chemical gating. Since then, the CaM role in gating has been confirmed by studies that tested it with a variety of approaches such as treatments with CaM-inhibitors, inhibition of CaM expression, expression of CaM mutants, immunofluorescent co-localization of CaM and gap junctions, and binding of CaM to peptides mimicking connexin domains identified as CaM targets. Our gating model envisions Ca^2+^-CaM to directly gate the channels by acting as a plug (“Cork” gating model), and probably also by affecting connexin conformation.

## 1. Direct Cell-To-Cell Communication

The neighboring cells of most tissues freely exchange small cytosolic molecules via cell-to-cell channels clustered at gap junctions. This form of direct cell–cell communication (cell coupling) provides a fundamental mechanism for coordinating and regulating a host of cellular activities in mature and developing organs [1,2,3,4,5,6,7]. Conversely, abnormal cell communication causes several diseases [8,9].

Each cell-to-cell channel is formed by the extracellular interaction of two hemichannels (connexons/innexons) that create a hydrophilic pathway spanning two apposed plasma membranes and a narrow extracellular space (gap). In turn, each connexon/innexon is an oligomer of six proteins (connexins/innexins) that span the membrane thickness and insulate the hydrophilic pore from the lipid bilayer and the extracellular medium. Gap junction channels are regulated by a gating mechanism sensitive to the cytosolic calcium concentration [Ca^2+^]_i_ [2,3,6,9,10].

## 2. Cell-To-Cell Uncoupling

In 1877, T.W. Engelmann made the startling discovery that cardiac cells become independent from adjacent injured cells [11], as he noticed that, in damaged cardiac muscle, unlike skeletal muscle, the injury potential soon vanishes, such that the electrical communication between healthy and damaged cardiac fibers ceases. This phenomenon, known as “healing over”, made him realize that there is a fundamental difference between cardiac and skeletal muscle cells. In his words: “*herzmuskelzellen leben zusamme und sterben einzeln* (cardiomyocytes live together and die alone)” [11]. Healing over, now known as “cell-to-cell uncoupling”, is present in all tissues with cells coupled by gap junction channels, and is mediated by the chemical gating mechanism [2,3,4,6,9,10,12,13,14].

### 2.1. Cytosolic Free-Calcium and Gap Junction Channel Gating

In 1965, Jean Délèze reported that cut cardiac fibers do not heal in the absence of extracellular calcium [12], suggesting for the first time a Ca^2+^-role in gap junction channel-gating. This observation was soon confirmed by evidence that electrical and dye couplings are lost with a [Ca^2+^]_i_ rise [13]. The Ca^2+^_i_ role in gating was proven by evidence that cell-to-cell uncoupling coincides with an increase in [Ca^2+^]_i_, monitored at the cell–cell contacts by aequorin luminescence [14]. The Ca^2+^_i_ role in gating was soon confirmed by many studies in both vertebrates and invertebrates [2,3,15,16,17].

#### [Ca^2+^]_i_ Effective on Channel Gating

Two early studies reported that only [Ca^2+^]_i_ in the high µM range causes cell-to-cell uncoupling [18,19]. However, numerous more recent reports have demonstrated that significantly lower [Ca^2+^]_i_, in the range of ~100 nM to low µM, are effective for channel gating. The effectiveness of low [Ca^2+^]_i_ was first published in studies on *Chironomus* salivary gland cells [20,21,22] and mammalian cardiac fibers [23,24].

In 1986, Noma and Tsuboi reported the effectiveness of [Ca^2+^]_i_ as low as 251 nM in cardiac cell-pairs [25,26]. Ten years later, Dekker and coworkers reported that the application of ionomycin and gramicidin to rabbit papillary muscle uncoupled the cells at [Ca^2+^]_i_ = ~685 nM or greater [27], and the same [Ca^2+^]_i_ uncoupled cells subjected to ischemia followed by reperfusion [27]. Low [Ca^2+^]_i_ were also effective in crayfish axons [28,29], rat lacrimal epithelial cells [30], Novikoff hepatoma cells [31,32], astrocytes [33,34,35], lens cultured cells [36], human fibroblasts [37], cultured cells expressing Cx43 [38] and pancreatic cells [39,40,41,42,43,44], among others.

In 1990, we studied the relationship between junctional electrical resistance (Rj), [Ca^2+^]_i_ and pH_i_ in crayfish septate axons uncoupled by intracellular acidification caused by superfusion with Na^+^-acetate (pH 6.3) [28]. With acetate, a [Ca^2+^]_i_ rise of approximately one order of magnitude from basal values of 100–300 nM greatly increased Rj [28]. The [Ca^2+^]_i_ and Rj time-courses coincided, while those of pH_i_ and Rj did not [28] (see in the following).

**Figure 1:**
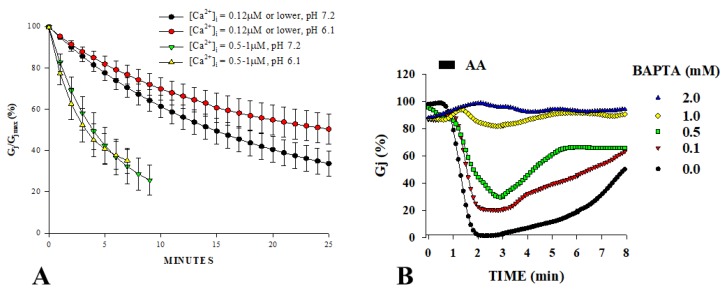
Junctional conductance (Gj) of Novikoff hepatoma cell-pairs expressing Cx43. (**A**). Cells dialyzed with patch-pipette solutions buffered for pH and Ca^2+^. With [Ca^2+^]_i_ = 0.12 µM or lower, Gj decreases to 40%–50% with τ’s of 35.2 and 22.3 min, at pH_i_ = 6.1 and 7.2, respectively—note that this is the normal Gj decay of whole-cell-clamped cells. With [Ca^2+^]_i_ = 0.5–1.0 µM, Gj decreases to ~25%, with τ’s of 5.9 and 6.2 min, at pH_i_ = 6.1 and 7.2, respectively. (**B**). In cell-pairs treated for 20 s with 20 µM arachidonic acid (AA), the rapid and reversible Gj drop is prevented by the buffering of Ca^2+^_i_ with low concentrations of BAPTA. Note that even a [BAPTA]_i_ as low as 0.1 mM has some inhibitory effect. (**A**,**B**) are adapted from Ref. [31] and [32], respectively.

In 1993, we determined more precisely the [Ca^2+^]_i_ effective on gating in Novikoff hepatoma cell pairs studied by double whole-cell patch-clamp [31,32]; these cell express connexin43 (Cx43). Ca^2+^-sensitivity was tested by monitoring the decay of junctional conductance (Gj) at different [Ca^2+^] at pH_i_ = 7.2 or 6.1. Gating was activated by [Ca^2+^]_i_ ranging from 500 nM to 1 µM, regardless of pH_i_ [31] (Figure 1A), proving that Cx43 channels are sensitive to [Ca^2+^]_i_ in the nM range and are insensitive to pH_i_ as low as 6.1, as long as [Ca^2+^]_i_ is kept at resting level with BAPTA in the patch pipettes [31].

The effectiveness of nM [Ca^2+^]_i_ was also demonstrated in Novikoff cells during brief (20 s) exposure to 20 µM arachidonic acid (AA) [32] (Figure 1B). AA caused rapid and reversible uncoupling that was completely prevented by Ca^2+^_i_-buffering with BAPTA in the patch pipette solutions (Figure 1B). Significantly, similar concentrations of EGTA, a less efficient Ca^2+^-buffer, were ten times less effective than BAPTA. AA (20 s exposure) had no effect on coupling in cells superfused with no-added-Ca^2+^ solutions (SES-no-Ca), suggesting that uncoupling resulted from Ca^2+^ entry [32]. In parallel experiments, [Ca^2+^]_i_ monitored with fura-2 increased with AA to 0.7–1.5 µM in cells with normal extracellular Ca^2+^ concentration ([Ca^2+^]) [32]. However, extensive AA treatments slowly uncouple Novikoff cells, even in Ca^2+^-free media, indicating that AA has also a slow, Ca^2+^-independent effect on gating [32], probably similar to that caused by anesthetics [45].

In cultured embryonic cells of chicken’s lens exposed to Ca-ionophores A23187 or ionomycin, [Ca^2+^]_i_ increased from ~110 to ~400 nM and cell–cell transfer of Lucifer Yellow was drastically reduced [36]. With the subsequent superfusion of salines containing EGTA with no added Ca^2+^, [Ca^2+^]_i_ dropped to ~260 nM and dye transfer resumed [36]. Similar nM [Ca^2+^]_i_ drastically decreased Gj in pancreatic β-cells, in which [Ca^2+^]_i_ was increased by lowering the temperature from 37 to 30 °C and increasing [Ca^2+^] from 2.56 to 7.56 mM [39].

[Ca^2+^]_i_ in the nM range was also effective in astrocytes co-injected with Lucifer Yellow and Ca^2+^, as the dye-transfer blockage was linearly related to [Ca^2+^]_i_ ranging from 150 to 600 nM [33]. Consistent with this is evidence that 20 mM BAPTA added to patch pipette solutions significantly increases coupling between astrocytes [34], suggesting that gating may even be sensitive to resting [Ca^2+^]_i_. Similarly, dye coupling was blocked in ionomycin-treated astrocytes by [Ca^2+^]_i_ = 500 nM [35], and comparable data were reported in lens-cultured cells [46]. In a recent study on murine Neuro-2a cells (N2a) expressing Cx43, exposure to ionomycin increased Ca^2+^ influx and reduced Gj by 95% [38]. [Ca^2+^]_i_ increased from ~80 to ~250 nM [38].

Based on these data, it is reasonable to believe that Ca^2+^_i_ is a fine modulator of cell communication via gap junction channels. Furthermore, evidence for the effectiveness of nanomolar [Ca^2+^]_i_ suggests the role of a Ca^2+^-modulated protein, calmodulin (CaM), as being the most likely candidate (see the following).

### 2.2. Intracellular pH and Channel Gating

In 1977, Turin and Warner reported that cytosolic acidification rapidly and reversibly uncouples *Xenopus laevis* embryonic cells [47,48], suggesting a pH_i_ role in channel gating. Soon after, uncoupling by lowered pH_i_ was confirmed in various vertebrate and invertebrate cells [19,49,50,51,52,53]. Curiously, however, pH_i_ has the opposite effect in cells expressing Cx36, as Gj increases with acidification and decreases with alkalinization [54]. Similarly, the alkalinization of insect cells to pH_i_ > 7.8 decreased Gj to a complete uncoupling, attributed to a [Ca^2+^]_i_ rise [49].

It is generally accepted that, in most cells, acidification causes uncoupling. However, pH_i_-sensitivity varies among cell types, and in most cases is related to the type of connexin expressed. In *Xenopus* oocyte pairs, for example, the rat liver connexin (Cx32) is much less sensitive than Cx38, the native oocyte connexin [55]. Delmar and coworkers tested the Gj sensitivity to pH_i_ in oocyte pairs expressing different connexins [56,57]. They demonstrated that Cx32 is the least sensitive (pKa ~6.5) and Cx50 the most sensitive (pKa ~7.2) connexin of this group; the other six connexins tested displayed pH-sensitivity in the following, decreasing order: Cx50, Cx46, Cx45, Cx26, Cx37, Cx43, Cx40 and Cx32 [58]. Other factors are also involved in determining pH_i_ sensitivity, because the same pH_i_ drops, attained by different procedures, have different effects on gating [28]. Furthermore, different cells expressing the same connexin have different pH_i_ gating sensitivities.

#### 2.2.1. Does pH_i_ Have a Direct Effect on Channel Gating?

Early data seemed to support a direct pH_i_ effect on connexin channels, although there were several inconsistencies [4,9,10,59]. Turin and Warner reported changes in coupling ratio (α) and pH_i_ in embryonic cells *Xenopus laevis*, but the pH_i_-α relationship was only plotted for the recoupling phase; thus, potential hysteresis could not be revealed [48]. Spray and coworkers, using recessed-tip pH-sensitive microelectrodes, reported a hysteresis in the pH_i_-α relationship, but interpreted it as an artifact caused by the CO_2_ effect on non-junctional membrane conductance (Gm); thus, they concluded that protons act directly on connexins (pKa = 7.3) [19]. However, subsequent work from the same group, using neutral-carrier, pH-sensitive microelectrodes, reported both a more pronounced hysteresis and a sensitivity to [H^+^]_i_ almost one order of magnitude lower (pKa = 6.5 versus 7.3) [60].

Campos de Carvalho and coworkers found that in crayfish axons, Gj, measured when pH_i_ drops, follows the same Hill relationship as Gj measured during pH_i_ recovery, but only with short acetate applications [61]. With longer acetate exposures, Gj recovered slowly and incompletely, in spite of the fact that pH_i_ recovered at normal rate [61]. Significant hysteresis in the Rj–[H^+^]_i_ relationship [28] and several other inconsistencies were also reported in sheep cardiac fibers [62] and crayfish axons [28].

**Figure 2:**
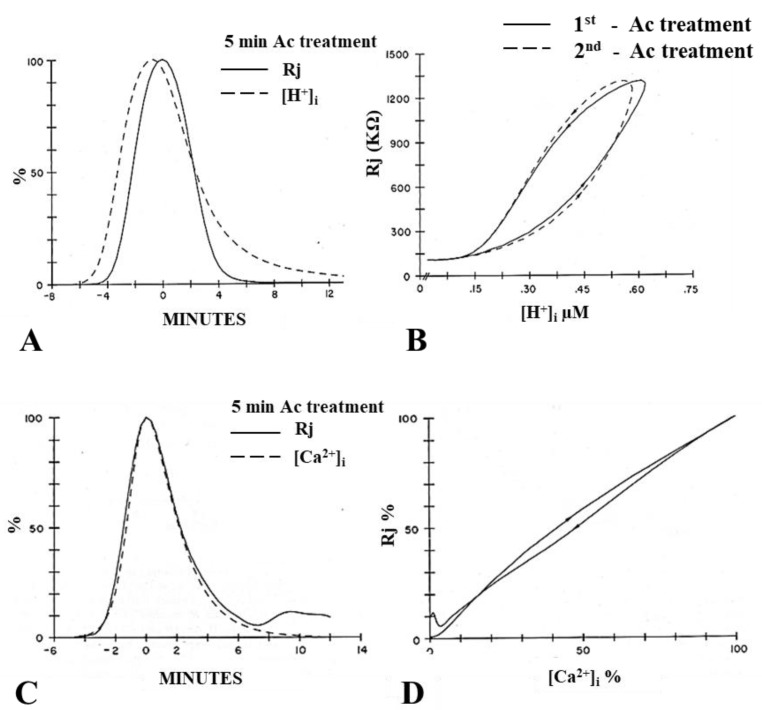
Percent change in junctional resistance (Rj) and [H^+^]_i_ in septal junctions of crayfish axons during cytosolic acidification with acetate solutions (Ac). (**A**). Plots of Rj–[H^+^]_i_ markedly differ in shape, and [H^+^]_i_ maxima precede Rj maxima by 40–90 s. (**B**). This results in marked curve hysteresis in the Rj–[H^+^]_i_ relationship, demonstrated in two different acetate superfusions (B: 1st and 2nd Ac treatments). In contrast, the Rj-[Ca^2+^]_i_ time courses (**C)** match extremely well in shape, peak time and magnitude. This results in negligible curve hysteresis in the Rj-[Ca^2+^]_i_ relationship (**D**). From Reference [28].

Channels made of Cx43 displayed a pKa of 6.7 in *Xenopus* oocyte pairs [58], while pH_i_ = 6.6 had only a small effect on coupling in Cx43-expressing mammalian heart fibers [53], and H^+^_i_ affected healing over in the heart only at pH_i_ < 5 [63]. In addition, internally perfused Cx43-expressing oocyte pairs were insensitive to pH_i_ [64], and Cx43-expressing Novikoff hepatoma cells were insensitive to pH_i_ = 6.1, as long as [Ca^2+^]_i_ was kept to resting levels with BAPTA in the patch pipettes (Figure 1) [31]. In crayfish axons, pH_i_ = 6.3 achieved by superfusion with acetate greatly increased Rj [28], while pH_i_ = 6.0 did not change Gj in internally perfused crayfish axons [65,66]. Note that, in crayfish axons, the Rj and [H^+^]_i_ time-courses markedly differ in shape, and [H^+^]_i_ maxima precede Rj maxima by 40–90 s (Figure 2A) [28]. In addition, media with pH as low as 5 do not affect the permeability of Cx32 hemichannels in liposomes [67], and several inconsistencies in the pH_i_/electrical-coupling relationship were described in insect gland cells [49].

In several studies, similar pH_i_ values attained by different procedures had different effects on coupling. In crayfish axons, Rj maxima obtained with slow acidification rates were three times greater than those with faster acidifications, although the same pH_i_ minima were reached [28]. The acidification of amphibian blastomeres to pH_i_ 6, induced by 100% CO_2_ exposure, brought Gj to zero, whereas a much greater degree of acidification was required to decrease Gj by the same amount when a low pH_i_ was achieved by HCl-injection, as pKa shifted from ~6.4 to ~5.7 [60]. Dunina-Barkowskaia and coworkers reported a similar discrepancy in uncoupling efficiency between cytosolic acidification induced by weak diffusible acids (CO_2_, acetate, etc.) and by cytosolic dialysis with a strong acid (HCl) [68].

Although most data suggest an indirect [H^+^]_i_ effect, in one study on hemichannels it appears that direct protonation may affect gating [69]. In this study, Cx46 hemichannels expressed in *Xenopus* oocytes and studied by patch clamp in excised patches were sensitive to pH_i_. Cx46 hemichannels closed rapidly and reversibly with short applications of low pH solutions, but channel gating was poorly reversible with longer treatments. This is unusual, because in intact cells uncoupling by acidification is always 100% reversible. A more recent study found that low pH may also act directly on Cx26 hemichannels [70]. This, however, contradicts earlier evidence for the role of aminosulfonates in the low-pH gating of Cx26 hemichannels [67,71,72].

#### 2.2.2. Does Ca_i_ Mediate the Effect of Acidification on Channel Gating?

The [H^+^]_i_–Rj and [Ca^2+^]_i_–Rj relationships were first studied in crayfish axons uncoupled by acetate superfusion [28]. Plots of the time-courses of Rj and [H^+^]_i_ markedly differ in shape, and [H^+^]_i_ maxima preceded Rj maxima by 40–90s (Figure 2A), resulting in significant curve hysteresis in the [H^+^]_i_–Rj relationship (Figure 2B). In contrast, the [Ca^2+^]_i_–Rj time-courses matched extremely well (Figure 2C), with no hysteresis (Figure 2D) [28], and different rates of acidification caused comparable increases in [Ca^2+^]_i_ and Rj [28]. These data clearly indicate that acidification-induced uncoupling is more closely related to [Ca^2+^]_i_ than [H^+^]_i_ [28].

**Figure 3:**
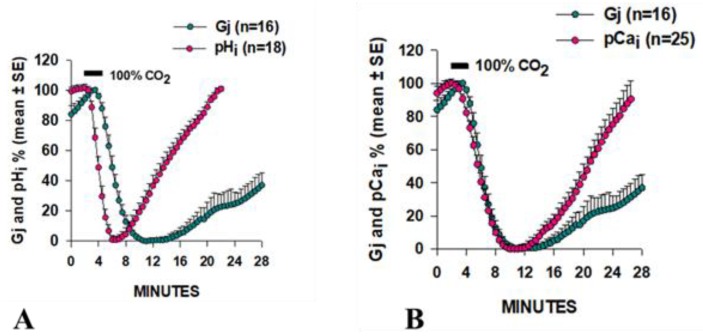
Relationship between junctional conductance (Gj) and either pH_i_ (**A**) or pCa_i_ (**B**) in *Xenopus* oocyte pairs (Cx38) superfused with salines gassed with 100% CO_2_. pH_i_ minima precede Gj minima by ~4 min (**A**), while Gj minima and pCa_i_ coincide (**B**). From Reference [73].

Similar results were obtained in *Xenopus* oocytes (Cx38) by monitoring Gj, with a double voltage clamp, as well as pH_i_ and pCa_i_ [73]. A drastic difference in time-course between Gj and pH_i_ was observed, as pH_i_ minima preceded Gj minima by ~4 min and pH_i_ recovered much faster than Gj (Figure 3A). In contrast, Gj minima and pCa_i_ minima matched well (Figure 3B) [73]. Plots of the time course of Gj and [H^+^]_i_ in *Xenopus* oocytes expressing Cx32 also showed marked differences [74].

Our data on *Xenopus* oocytes (Cx38) intracellularly buffered with BAPTA are in agreement with evidence that a low pHi uncouples cells by increasing [Ca^2+^]_i_, as they greatly inhibited the effect of acidification on Gj [73]. A similar inhibition occurred in oocytes previously injected with ruthenium red [73]. Since ruthenium red blocks the mitochondrial Ca^2+^-uniporter [75], acidification is likely to increase [Ca^2+^]_i_ by releasing it, at least partially, from the mitochondria. A low-pH_i_-induced Ca^2+^-release from the endoplasmic reticulum (ER) is also likely to be involved, as indicated by our study on crayfish axons uncoupled by a low pH_i_ in the presence of caffeine or ryanodine [59] (see the following). 

To understand the mechanism by which low pH_i_ causes a [Ca^2+^]_i_ rise, we tested drugs that affect Ca^2+^ release from internal stores (caffeine and ryanodine) on crayfish axons, as well as treatments that cause Ca^2+^ entry [29]. A large increase in Rj and [Ca^2+^]_i_ maxima resulted from the addition of caffeine to acetate solutions, whereas a substantial drop in Rj and [Ca^2+^]_i_ maxima was seen when the acetate-caffeine treatment was preceded by caffeine pretreatment, suggesting that the caffeine pretreatment depleted the Ca^2+^-stores. In contrast, ryanodine always caused a significant decrease in Rj and [Ca^2+^]_i_ maxima [29]. Acetate-induced increase in Ca^2+^ entry was excluded because Ca^2+^-channel blockers were ineffective [59]. Acetate solutions containing [Ca^2+^] as high 40 mM were also ineffective [59], further confirming that Ca^2+^ entry does not play a role in acetate-induced [Ca^2+^]_i_ increase.

Therefore, we have concluded that the effect of low pH_i_ on gating is mediated by Ca^2+^_i_ [9,10]. Indeed, most compelling in this regard are our data on Novikoff cells, in which acidification to pH_i_ = 6.1 had no effect on gating as long as [Ca^2+^]_i_ was carefully buffered to resting levels by BAPTA in the patch pipettes [4,31] (see previous).

## 3. Calmodulin Role in Cell-To-Cell Channel Gating

In 1981, Johnston and Ramón reported that internally perfused crayfish lateral giant axons do not uncouple with either a high [Ca^2+^]_i_ or low pH_i_ solution [65]. Based on these data, confirmed by Arellano and coworkers [66], they suggested that a soluble intermediate mediates the Ca^2+^/H^+^-induced channel gating. In the same year, we proposed the role of calmodulin (CaM) in gap junction channel gating [76,77]. The CaM role was also supported by evidence for CaM binding to Cx32 and gap junction fragments from crayfish hepatopancreas [78,79]. Over the years, the CaM role in gating has been confirmed by data generated from various approaches such as the application of CaM blockers, inhibition of CaM expression, overexpression of CaM mutants, co-localization of CaM and gap junctions, and in vitro evidence for CaM binding to connexins or synthetic connexin peptides mimicking CaM-binding sites, among others [9,10,80].

In addition to gap junction channels, CaM-regulated channels include voltage-gated Ca^2+^ channels (VGCC, CaV), Na^+^ channels (VGSC, NaV), K^+^ channels (VGPC, KV), small conductance Ca^2+^-activated K^+^ channels (SK), inwardly rectifying K^+^ channels (Kir, IRK), cyclic nucleotide-gated channels (CNG), ryanodine receptors (RyR) and transient receptor potential channels (TRP), among others [81,82,83,84]. CaM also gates the water channel aquaporin-0 (AQP0), also known as the eye lens protein MIP26 [85,86,87,88,89,90,91,92].

### 3.1. CaM Inhibitors Prevent Cell-To-Cell Uncoupling

**Figure 4:**
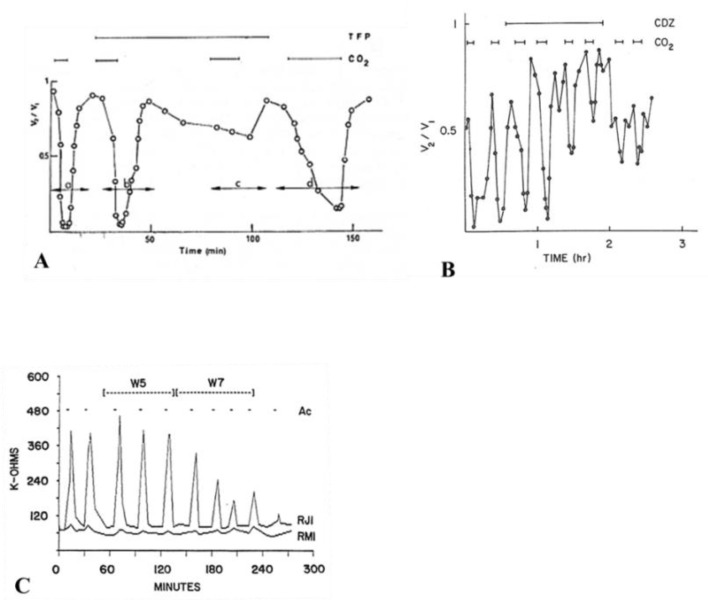
Electrical coupling of neighboring *Xenopus* embryonic cells at morula stage (**A**,**B**), monitored by measuring the coupling ratio (V_2_/V_1_). In the absence of the CaM-inhibitor trifluoperazine (TFP), 100% CO_2_ reduces V_2_/V_1_ to nearly zero, while, with 5 µM TFP, CO_2_ has a minimal effect (**A**). The effect of TFP is slowly reversible (**A**). The most specific CaM-blocker, calmidazolium (CDZ), also inhibits the effect of CO_2_ (**B**). With 100 nM [CDZ], 100% CO_2_ causes V_2_/V_1_ to only drop to 0.5–0.7 (**B**). Another CaM inhibitor (W7, 100 µM) also effectively inhibits Ac-induced Rj rise in crayfish axons (**C**), while its control (W5) is totally ineffective (**C**). A, B and C, from References [76,93,94], respectively.

In 1981, we first reported the CaM role in chemical gating, based on the ability of trifluoperazine (TFP), a CaM blocker, to prevent CO_2_-induced uncoupling of *Xenopus* embryonic cells (Figure 4A) [76,77]—indeed, this was the first example of CaM participation in the gating mechanism of membrane channels. In this study, we monitored the electrical coupling of *Xenopus* embryonic cells (morula stage) by measuring the coupling ratio (V_2_/V_1_), whose drop reflects electrical uncoupling. In controls, exposure to 100% CO_2_ always reduced V_2_/V_1_ to nearly zero, while after 45–60 min treatment with 5 µM TFP, CO_2_ had a minimal effect on V_2_/V_1_ (Figure 4A). The TFP effect was reversible, but full recovery of gating efficiency was very slow (Figure 4A). 

In a subsequent study, calmidazolium CDZ), the most specific CaM blocker, also prevented uncoupling in *Xenopus* embryonic cells [93]. With 100 nM CDZ, 5 min exposures to 100% CO_2_ caused V_2_/V_1_ to drop to 0.5–0.7 (Figure 4B)—note that, in the absence of CDZ, CO_2_ caused V_2_/V_1_ to drop to nearly zero (Figure 4B). CDZ also improved coupling, as V_2_/V_1_ reversibly increased at rest from ~0.6 to ~0.8 (Figure 4B) [93].

The CaM blocker W7 also inhibited uncoupling of crayfish axons superfused with acetate-containing salines (pH = 6.3) [94]. W7 (100 µM) strongly inhibited the Rj rise within 15–20 min of treatment (Figure 4C), while its control (W5) was totally ineffective (Figure 4C)—recovery of normal uncoupling efficiency was slow and usually incomplete.

Studies on cardiac [95,96] and lens [46,97] cells reported similar results. In pairs of guinea pig ventricular myocytes, one of which was voltage clamped, where Gj was monitored after perforation of the partner-cell’s non-junctional membrane, the gating sensitivity to Ca^2+^ increased from pCa 5.7 to pCa 7 when the cells were perfused with 10 µM CaM. Significantly, W7 (but not W5) prevented uncoupling [98]. In some cases, CaM inhibitors have also been reported to increase coupling (Figure 4B) [93,99].

Lurtz and Louis tested CDZ in HeLa cells stably transfected with Cx43 [46]. Exposure to 1 μM ionomycin in the presence of increased [Ca^2+^]_o_ increased [Ca^2+^]_i_ from ~110 to ~620 nM and blocked the cell-to-cell diffusion of AlexaFluor594. The block was prevented by pre-incubation with 10 µM CDZ. Recently, CDZ was also effective in murine Neuro-2a cells (N2a) expressing Cx43 [38]—exposure to 1 µM ionomycin increased [Ca^2+^]_i_ from ~80 to 250 nM and reduced Gj by ~95% in 15 min; the Gj drop was prevented by bathing the cells in Ca^2+^-free solutions or pretreating them with 2 µM CDZ [38].

Paradoxically, CaM inhibitors uncoupled insect epidermal cells [100] and Hansen cells of the guinea pig cochlea [101], which express Cx26 and Cx30 [102]. Perhaps, in these cells, inhibition of the CaM–connexin interaction, or CaM release from connexins, causes channel closing rather than opening. Significantly, this also occurs in Cx32 or Cx43 hemichannels, as W7 prevents the Ca^2+^-induced hemichannel-opening [103,104].

### 3.2. Inhibition of CaM Expression Prevents Cell-To-Cell Uncoupling

**Figure 5:**
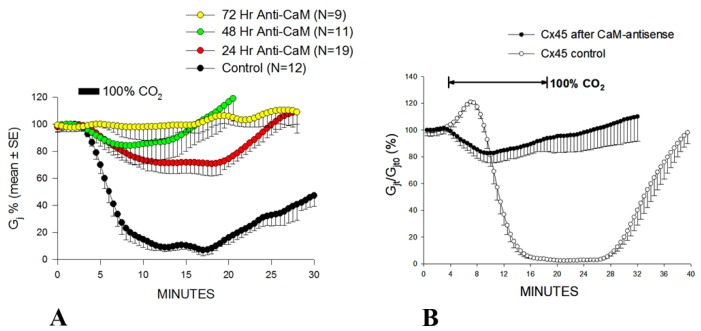
A. Junctional conductance (Gj), monitored in *Xenopus* oocyte pairs (Cx38), exposed to 100% CO_2_. Oocytes in which CaM expression is inhibited by the injection of antisense oligonucleotides progressively lose uncoupling efficiency (**A**). The CO_2_ effect on Gj is reduced by ~60%, ~76%, ~93% in 24, 48 and 72 h, respectively (**A**). (**B**). Gj monitored in oocyte pairs expressing Cx45 during CO_2_ exposure. The inhibition of CaM expression greatly reduces the CO_2_ sensitivity of Cx45 channels—with 15 min application of CO_2_, Gj reversibly drops by only ~17%, while in controls it drops to 0% (**B**). The Gj rise that precedes the Gj drop is absent with the inhibition of CaM expression (**B**). (**A**,**B**) from References [73,106], respectively.

The CaM hypothesis was also tested by monitoring the effect of exposing to 100% CO_2_ *Xenopus* oocyte pair (Cx38) on Gj, in which CaM expression was inhibited by the previous injection of oligonucleotides antisense into the two CaM mRNAs expressed in oocytes [73]. Antisense oligonucleotides caused a progressive loss of uncoupling efficiency, starting 24 h post-injection (Figure 5A) [73]. Gating sensitivity to CO_2_ partially recovered with CaM injection [73].

Antisense oligonucleotides had the same effect in oocytes expressing heterotypic mutant-Cx32 [105] or Cx45 (Figure 5B) channels [106]. Homotypic Cx32 channels (32–32) have low CO_2_ sensitivity (Figure 8) [55]. In contrast, certain heterotypic mutant-32 channels are significantly more sensitive to CO_2_ (Figure 9). Following inhibition of CaM expression, in mutant-32 channels CO_2_ had a minimal effect on Gj [105]. In oocytes expressing Cx45, Gj drops to nearly zero with 15 min CO_2_ application (Figure 5B) [106]. In contrast, with the inhibition of CaM expression, 15 min CO_2_ applications caused Gj to drop by only 17.3% (Figure 5B) [106].

### 3.3. A CaM Mutant with Higher Ca^2+^-Sensitivity Greatly Enhances Gating Sensitivity

**Figure 6:**
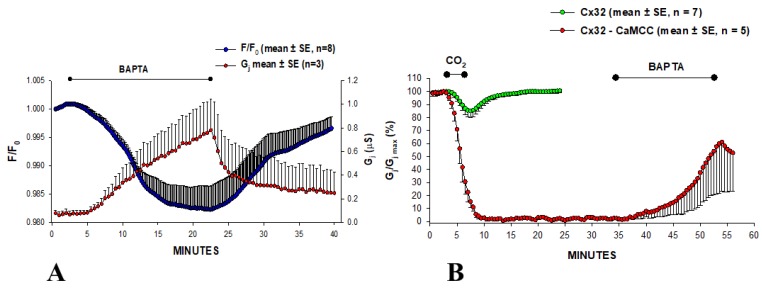
Junctional conductance (Gj) and Ca^2+^_i_ (**A**) monitored in *Xenopus* oocyte pairs expressing Cx32. In oocytes expressing CaMCC, Gj is very low, but it dramatically and reversibly increases when [Ca^2+^]_i_ is lowered with 180 µM BAPTA superfusion (A). This indicates that CaMCC greatly increases Ca^2+^-gating sensitivity, such that even basal [Ca^2+^]_i_ affect gating. This was confirmed by testing the effect of CO_2_ (**B**). With 3 min exposure to 100% CO_2_, Gj rapidly drops to zero, whereas in controls it decreases by only ~15% (B); Gj remains at 0 indefinitely, but recovers (reversibly) with 180 µM BAPTA application (**B**). Adapted from Reference [107].

The effect of overexpressing the CaM mutant CaMCC on chemical- and Vj-gating was tested on oocytes expressing Cx32 channels [107,108]. In CaMCC, the NH_2_-terminal EF-hand pair (res. 9–76) of CaM is replaced by a duplication of the COOH-terminal pair (res. 82–148). Since the Ca^2+^ affinity constant of the COOH-terminal EF-hand pair is almost one order of magnitude greater than that of the NH_2_-terminal pair [109], we felt that expression of CaMCC might enhance chemical gating sensitivity. Indeed, in oocytes expressing CaMCC and Cx32 Gj was very low, but dramatically and reversibly increased (Figure 6A) when [Ca^2+^]_i_, monitored with Calcium Green-1, was lowered by 180 µM BAPTA superfusion—note the reversible drop in F/F_0_ (Figure 6A). This suggests that CaMCC increases the Ca^2+^ sensitivity of gating to such an extent that even resting [Ca^2+^]_i_ affect gating. This was confirmed by testing the effect of CO_2_. With 3 min exposure to 100% CO_2_, Gj rapidly dropped to zero, whereas in controls it decreased by only ~15% (Figure 6B). Gj remained nearly at zero indefinitely, but started recovering (reversibly) with a superfusion of 180 µM BAPTA (Figure 6B). Significantly, CaMCC was effective only when it was expressed before Cx32. This indicates that CaMCC, and by extension native CaM, binds to Cx32 before connexon assembly, further supporting the idea that CaM is an integral subunit of the connexon. The intimate CaMCC–Cx32 relationship was confirmed by a large reduction in Vj sensitivity [107].

CaM mutants lacking one or more of the four high-affinity Ca^2+^-binding sites were also tested [10]. In these mutants, glutamates (E) important for Ca^2+^ binding were replaced by alanines (A) in CaM’s EF–hand domains—these mutations greatly decreased the Ca^2+^ affinity of the Ca^2+^-binding EF–hand loops [110]. The expression of CaM_1,2,3,4_ (E32A, E68A, E105A, E141A) or CaM_1,2_ (E32A, E68A), preceding Cx32 expression, strongly inhibited the formation of functional Cx32 channels, whereas the expression of CaM_3,4_ (E105A, E141A) had no effect [10]. Evidence that CaM_3.4_, unlike CaM_1,2_, enables an almost normal gap junction expression, suggests that N-lobe’s Ca^2+^-activation is needed for gap junction formation. The effectiveness of CaM_1,2,3,4_ in competing against CaM wild-type confirms Ca^2+^-independent CaM-binding to connexins [111,112]. Furthermore, the observation that CaM_1,2_, but not CaM_3,4_, prevents channel formation, further indicates that normal Ca^2+^-affinity of CaM’s N-lobe is needed. Indeed, the CaM–connexin interaction was reported to be relevant to connexin oligomerization into connexons, as in an in vitro cell-free synthesis system the formation of Cx32’s hexameric hemichannels was reversibly inhibited by a CaM-binding synthetic peptide and W7 [113]. Removal of the CaM-binding site at the COOH-terminus domain (CT1) impaired connexon formation and caused an accumulation of intermediate connexin oligomers [113]. Recently, these data were confirmed for Cx36 [114]. However, these data contradict our evidence for normal gap junction formation in oocytes expressing CT-truncated Cx32 [74,115,116] or Cx40 [117] channels.

### 3.4. Do CaM-Activated Enzymes Play a Role in Gating?

Ca^2+^/CaM kinase II has been shown to phosphorylate Cx32, but only in isolated junctions as intact hepatocytes exposed to the Ca^2+^-ionophore ionomycin did not became phosphorylated [118]. The activation of Ca^2+^/CaM kinase II increased Gj in mouse astrocytes [119] and goldfish Mauthner cells [120], but whether mechanisms other than connexin phosphorylation are involved is unclear.

We have tested the potential role of inhibitors and/or activators of a number of enzymes [10]. None of them, however, significantly changed Gj or CO_2_-induced channel gating [10]. The potential role of Ca^2+^-activated proteases is unlikely because proteolysis would be irreversible and the recovery rate from Ca^2+^-induced uncoupling is much more rapid than the connexin’s turnover time (half-life = ~3 h) [121].

The possibility that gating by raised [Ca^2+^]_i_ results from the activation of Protein kinase C (PKC) or CaM-kinase II was tested in lens epithelial cells by raising [Ca^2+^]_i_ via Ca^2+^-ionophore or ATP treatment [46,122]. Cell-to-cell transfer of AlexaFluor594 decreased with a rise in [Ca^2+^]_i_ and was prevented by CDZ treatment before ionomycin addition, but not by inhibitors of PKC or CaM-kinase II. Significantly, in HeLa cells transfected with a CT-truncated Cx43 mutant (Cx43-D257), the drop in coupling caused by a [Ca^2+^]_i_ rise to ~300 nM was similarly prevented by CDZ [122]. This not only confirms that Ca^2+^_i_ regulates Cx43 channel gating in a CaM-dependent manner, but also proves that gating does not require the Cx43’s CT domain. Evidence that CT does not play a role in Cx43 channel gating is also supported by recent data reporting that CT-deletion at res. 257 does not affect the gating sensitivity of Cx43 channels to a rise in ionomycin-induced [Ca^2+^]_i_ [123].

### 3.5. Co-Localization of Cam and Connexins

**Figure 7:**
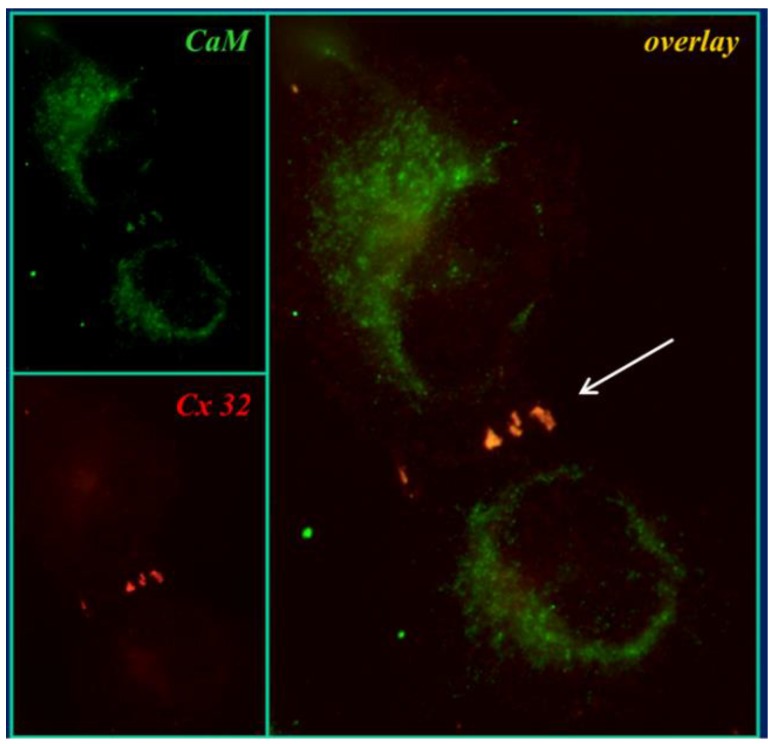
Immunofluorescence images of HeLa cells stably transfected with Cx32. CaM and Cx32 co-localize at the cell–cell contact area (overlay). Adapted from Reference [108].

In 2000, we tested the direct CaM–connexin interaction by immunofluorescence microscopy [107,108]. In HeLa cells expressing Cx32, CaM and Cx32 co-localized in punctated or linear areas of cell–cell contact (Figure 7, overlay’s arrow) as well as in few cytoplasmic spots [107,108]. CaM-Cx32 co-localization was also confirmed in cryosectioned mouse liver [108]. Similar results were obtained with Cx43 and Cx37 (Sotkis and Peracchia, unpublished data), as well as with Cx50 [124,125] and Cx36 [114]. These results confirm immuno-electron-microscopy data that demonstrated CaM binding to gap junctions of myocardial cells stained in frozen thin sections with colloidal gold-labeled CaM [126].

The direct CaM–Cx32 interaction was also demonstrated by confocal fluorescence microscopy in HeLa cells, expressing Cx32 bound to the green fluorescent protein (Cx32-GFP) and CaM bound to the red fluorescent protein (CaM-RFP) [9,10,108]. In these samples, however, CaM and Cx32 only co-localized at cytoplasmic spots, as these cells did not form junctional plaques. The absence of junctional plaques may be due to steric hindrance, as the large size of the two fusion proteins may hinder connexin oligomerization into connexons. Similar results were obtained with Cx32 linked to the cyan fluorescent protein (Cx32-CFP) and CaM linked to the yellow fluorescent protein (CaM-YFP) [10,108].

Recently, the CaM–Cx45 interaction was directly visualized in a remarkable study that monitored it in living cells by Bioluminescence Resonance Energy Transfer (BRET) [127]; the interaction was Ca^2+^-dependent and blocked by W7. The role of CL2’s CaM-binding site (res. 164–186; Figure 11) was confirmed by its high-affinity interaction (K_d_ = ~5 nM) with a peptide mimicking the CL2 domain, monitored by fluorescence-labeled CaM [127]. Note, however, that there is evidence for both Ca^2+^-dependent and Ca^2+^-independent CaM binding to the CL2 domain of Cx32, Cx35, Cx45, and Cx57 [111,112]. The Ca^2+^-independent CaM–CL2 interaction confirms previous evidence that CaM is anchored to connexins at resting [Ca^2+^]_i_ (~50 nM) [9,10,16,107,108,127].

### 3.6. Connexin Domains Potentially Relevant to Chemical Gating

**Figure 8:**
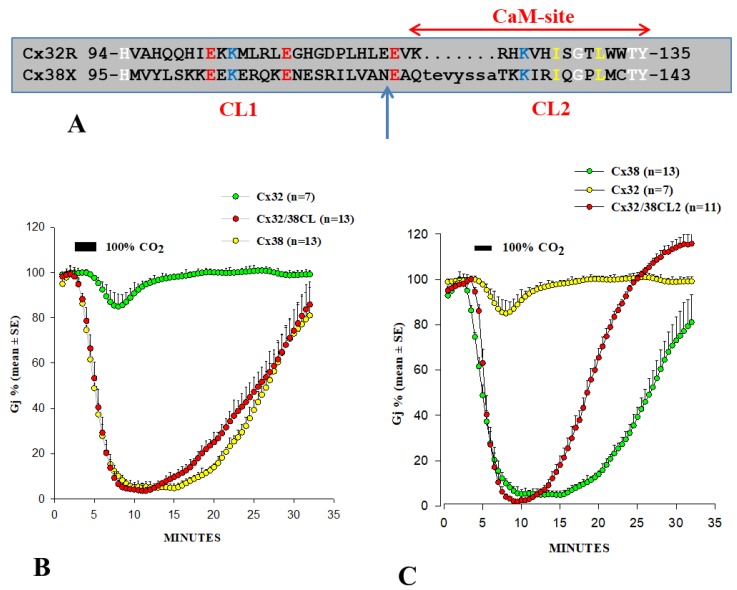
(**A**) Cytoplasmic loop (CL) sequences of rat Cx32 and *Xenopus* Cx38. (**B**,**C**) Junctional conductance (Gj) monitored in *Xenopus* oocyte pairs expressing Cx32, Cx38 or Cx32/38 chimeras. Channels made of Cx32/38CL (Cx32’s CL replaced by that of Cx38) reproduce almost exactly the gating efficiency of Cx38 channels in magnitude and rate (**B**). Similar results were obtained with channels made of Cx32/38CL2 (Cx32’s CL2 replaced by that of Cx38, C), although Gj recovered faster. Note that CL2 contains the CaM binding site (**A**). The postulated CL1–CT1 interaction (see text) could explain the faster recovery of Cx32/38CL2 channels (**C**). B and C adapted from References [55,128], respectively.

In several studies, we have attempted to identify connexin domains relevant to chemical gating [55,74,116,128,129,130]. In 1996, we tested the CO_2_ sensitivity of channels made of Cx32 and Cx38 chimeras and mutants in oocytes. As previously mentioned, Cx32 and Cx38 make channels that are at the opposite end of the spectrum in terms of CO_2_ sensitivity (Figure 8) [55,128].

We focused first on the cytoplasmic loop (CL) of Cx32 and Cx38 (Figure 8A). Channels made of Cx32/38CL (Cx38’s CL replacing that of Cx32) reproduced the chemical gating efficiency of Cx38 channels in uncoupling magnitude and both uncoupling and recoupling rates almost exactly (Figure 8B). In contrast, channels made of Cx32/38NT (Cx38’s NT replacing that of Cx32) behaved closer to Cx32 channels [55]. Cx38 channels are more sensitive to fast-Vj gating than those of Cx32 [55]. Of the two chimeric channels, Cx32/38CL channels displayed fast-Vj sensitivity, similar to that of Cx38 channels, whereas Cx32/38NT channels displayed very low Vj sensitivity. The data suggest that CL plays a major role in both chemical gating and fast-Vj gating sensitivities [55].

To further identify the most relevant CL domains, we tested Cx32/Cx38 chimeras in which either the first half (CL1) or the second half (CL2) (Figure 8A) of Cx38’s CL replaced those of Cx32 [128]. The Cx32/Cx38CL2 channels (Cx32 with CL2 of Cx38) were like Cx38 in CO_2_ sensitivity, although Gj recovered faster than in Cx38 channels (Figure 8C), but similar to Cx32 in fast-Vj gating sensitivity. Cx32/Cx38CL1 (Cx32 with CL1 of Cx38) did not express channels. These data indicate that CL1 and CL2 contain domains that are relevant for fast-Vj gating and chemical gating, respectively [128]. In fact, CL2’s relevance to chemical gating is consistent with evidence for the presence of a CaM binding-site at CL2 (Figure 11) of Cx32, Cx35, Cx45 and Cx57 [111,112] (Table 1), as well as that of Cx43, Cx50 and Cx44 [80]. CaM binding sites at CL2 are also present in Cx26, Cx31, Cx33, Cx36, Cx37, Cx40 and Cx46 (Figure 11).

The faster recovery of Cx32/Cx38CL2 channels (Figure 8C) might result from a potential CL1-CT1 interaction, the idea being that in open channels CL1 might be bound to CT1 (see in the following). If so, the presence of the Cx32’s CL1 domain in Cx32/Cx38CL2 channels may enable a fast channel reopening due to the rapid recovery of the CL1–CT1 interaction, following the unbinding of the CaM’s lobe from the gating site (see in the following: Ca–CaM–Cork gating).

**Figure 9:**
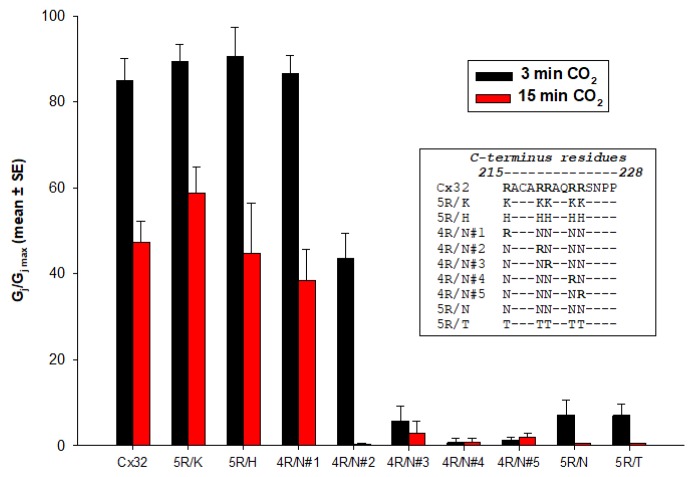
Normalized junctional conductance (G_j_/G_j max_) monitored in *Xenopus* oocyte pairs expressing Cx32 or Cx32’s CT1 mutants. Channels made of Cx32 mutants in which two or more CT1-arginines (R215, R219, R220, R223 and R224) are replaced by neutral-polar residues (asparagine, N or threonine, T) are much more sensitive to CO_2_ than controls. In contrast, channels in which the R-residues are replaced with another basic residue (lysine, K) or histidine (H), as well as those with preserved R215 (4R/N#1), behave like Cx32 wild-type channels. From Reference [116].

The potential relevance of Cx32’s CT was tested by constructing a chimera in which Cx32’s CT was replaced with that of Cx38 (Cx32/Cx38CT). However, this chimera did not express functional channels. Therefore, we focused instead on the potential role of a domain at the NH_2_-end of CT (CT1), which has been identified as a CaM binding site in Cx32 [131,132], and indeed binds CaM [132,133]. Various mutations at Cx32’s CT1, and CT deletions, yielded interesting yet puzzling results [74,116]. Although much of Cx32’s CT is irrelevant to chemical gating, as 84% CT deletion (Cx32–D219) does not affect CO_2_ sensitivity [74,134], basic CT1 residues appear to contribute to the low CO_2_ sensitivity of Cx32 channels. This is suggested by the functional behavior of mutants in which five CT1 arginines (R215, R219, R220, R223 and R224) were replaced by neutral-polar residues (asparagine, N or threonine, T), another positively charged residue (lysine, K), histidine (H) (Figure 9) or the acidic residue glutamate (E) [74,116]. 5R/N and 5R/T channels were much more sensitive to CO_2_ than Cx32 channels (Figure 9) with both full-length Cx32 (5R/N) and Cx32 with CT deleted at residue 225 (D225–5R/N). In contrast, 5R/K and 5R/H channels were as sensitive to CO_2_ as Cx32 wild-type (Figure 9) [116]. CO_2_ sensitivity is strongly inhibited by R215 and mildly by R219, whereas R220, R223 and R224, may slightly increase, rather than inhibit, CO_2_ sensitivity (Figure 9) [116]; thus, R215 (and to a lesser extent R219) are most relevant in inhibiting CO_2_ gating sensitivity [74,116]. The mutation of the five R residues to glutamate (5R/E), tested in heterotypic 5R/E-32 channels, increases the CO_2_ gating sensitivity even more [105,135]. Therefore, the mutants ranked as follows in terms of CO_2_ sensitivity: 5R/E > 5R/N > Cx32wt.

These data were puzzling because CT1 (res. 208–227) was reported to be a CaM binding domain [132,133], and so its basic residues are expected be relevant for CaM binding. The greater CO_2_ sensitivity of 5R/N and 5R/E mutants seems to indicate that the greater the reduction in CT1’s CaM-binding affinity, the greater the CO_2_ channel gating sensitivity. Indeed, presently we doubt that the CT1’s CaM binding is relevant to chemical gating, because CT-deleted Cx32 mutants (Cx32–D219) generated channels indistinguishable from Cx32 wild-type in CO_2_ gating sensitivity [74].

**Figure 10:**
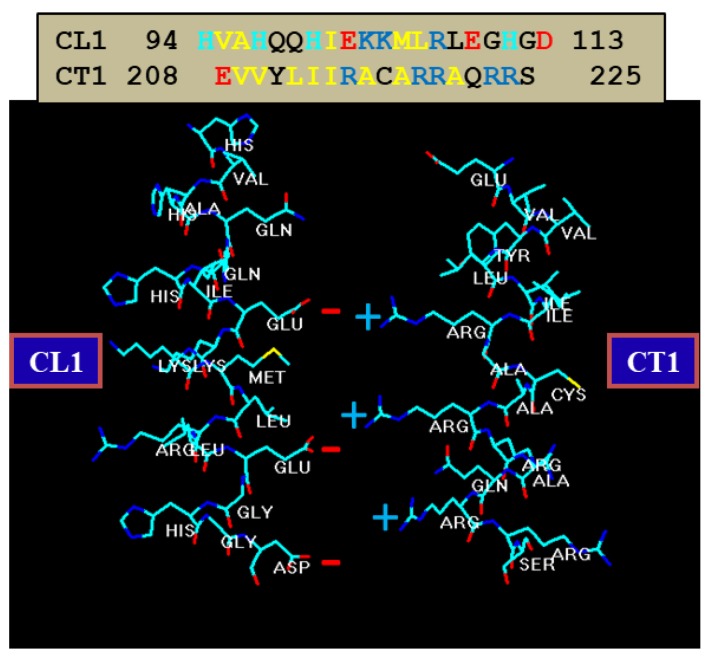
Alpha-helical structure of Cx32’s CL1 and CT1 domains. CL1 is the only cytoplasmic domain rich in negative charges—it also contains positive charges, but they are on opposite sides of the helix. Our hypothesis is that, in coupled conditions, CL1 interacts with CT1. In CL1, acidic residues (E102, E109 and D113) and hydrophobic residues (M105 and L106) are on the same side of the helix. The idea is that, with uncouplers, a CaM lobe accesses the gating site by breaking the CL1–CT1 interaction (Ca–CaM–Cork model).

A possible role of CT1 in channel regulation is its potential binding to CL1 (Figure 10)—this binding, if present, may need to break to allow CaM to bind to CL2. Perhaps, in the absence of CT1’s R residues, this domain does not bind to CL1. This is further suggested by data showing that heterotypic channels made of Cx32 and the mutants 5R/E or 3R/N–ML/NN (R215, 219 and 220 replaced by N, and M105 and L106 replaced by N) are very sensitive to CO_2_ [105,135]. Therefore, it could be that the postulated CL1–CT1 binding requires both electrostatic and hydrophobic links—note that the hydrophobic residues, M105 and L106, and the acidic residues E102, E109 and D113, which are expected to interact with CT1’s R residues, are on the same side of the alpha-helix (Figure 10) [9].

While the Cx32’s CT domain is unlikely to play a role in chemical gating [74,130], it seems to be relevant to gap junction assembly. Indeed, in human pancreatic and prostatic cancer cells, CT-deleted Cx32 (at res. 220) assembles into smaller gap junctions [136].

### 3.7. CaM Binding to Connexins and Connexin Peptides—Relevance to Channel Gating

Hertzberg and Gilula first demonstrated the ability of CaM to bind to Cx32 in gel overlays [78]. CaM-binding to Cx32 and Cx32 fragments was soon confirmed by several teams [79,132,137]. CaM–Cx32 interaction was also demonstrated by evidence that CaM prevents both Cx32 proteolysis by m-calpain [138] and Cx32 phosphorylation by EGF receptor tyrosine kinase [139]. CaM–connexin interaction is also indirectly suggested by evidence for its participation in Cx32 oligomerization into connexons in vitro [113].

In 1988, we first identified two CaM binding sites in Cx32: one at NT (res. 15–27; RHSTAIGRVWLSV) and one at CT1 (re. 209–221; EVVYLIIRACARR) [131]. Török and coworkers tested the CaM-binding to peptides matching the sequences of Cx32’s NT and CT1 domains by the fluorescent CaM derivative TA-CaM (2-chloro-(t-amino-Lys7s)- [6-[4-(7V,7V-diethylamino)-phenyl]-l,3,5-triazin-4-yl]calmodulin) [140] and equilibrium fluorescence methods [132]. Both peptides bound TA–calmodulin in Ca^2+^-dependent manner. The dissociation constants (kDa) of TA–CaM binding to the NT and CT1 peptides were 27 nM and 1.2 µM, respectively. In a subsequent study, Dodd and coworkers tested the lobe-specific interactions of CaM with Cx32 peptides by stopped flow kinetics, using Ca^2+^-binding-deficient CaM mutants [133]. Peptides matching the NT domain Cx32 (res. 1–22) bound to both NH_2_- and COOH-terminal CaM lobes (N- and C-lobes), but bound with higher affinity to the C-lobe. In contrast, peptides corresponding to the CT1 domain (res. 208–227) interacted with either CaM lobe, but with only one lobe at a time [133].

CaM binding to Cx32’s CT1 was confirmed by testing the interaction with Isothermal Titration Calorimetry (ITC) and Nuclear Magnetic Resonance (NMR) [141]. However, in this study, which used a longer amino acid chain (res. 217–283), both CaM lobes bound to the peptide. Recently, the Cx43’s CT res. K264–T290 has also been found to bind CaM [142]. However, none of these Cx43’s CT CaM-binding sites are relevant for chemical gating because Cx43’s CT-deletion at res. 257 does not affect the gating sensitivity to the ionomicin-induced increase in [Ca^2+^]_i_ [123].

CaM also binds to the CT1 domain of mouse Cx34.7 [114,143]. The interaction of CaM with Cx36’s CT1 was further tested by NMR; this study demonstrated that the CaM binds to a synthetic peptide mimicking the CT1 site in its typical compact state to an eight-residue domain (mostly hydrophobic) spanning residues W277–V284 [114]. The Cx36–CaM complex preceded the formation of Cx36 gap junction plaques and enabled dye coupling [114]. Significantly, CaM inhibitors, or the mutation of the residue W277, relevant for CaM binding to Cx36, inhibited dye coupling [114]. Evidence for CaM–Cx36 interaction before plaque formation confirms the role of CaM in gap junction formation [113].

The original study of Burr and coworkers reported that the dissociation constants (kDa’s) of the high affinity sites range from 11 to 72 nM, and K_1/2_’s for Ca^2+^ range from 3 to 5 µM [143]. Ca^2+^–CaM sensitivity in the µM range is higher than expected, but consistent perhaps with evidence that Cx36 channels are insensitive to pH_i_ as low as ~6.5 [54]. In these Cx36-expressing cells, uncoupling occurred with alkalinization; this may result from a high-pH_i_-induced [Ca^2+^]_i_ rise [144]. In fact, the cytosolic alkalinization of insect cells increased [Ca^2+^]_i_ and caused uncoupling at pH_i_ > 7.8 [49].

**Figure 11:**
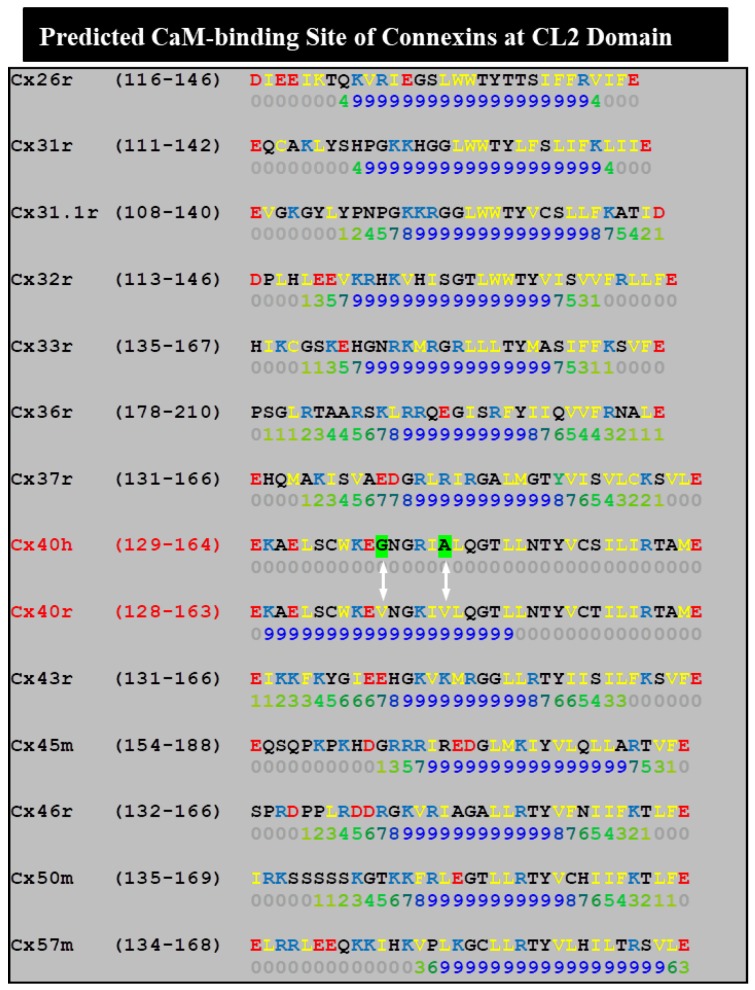
Analysis of the CL2 domain as a potential CaM-binding site. The CaM sites were identified by a program that predicts them on the scale 0–9. In all but human-Cx40 (Cx40h), the CL2 domain contains a CaM site. CaM-binding to the CL2 domains of Cx32, Cx35, Cx43, Cx44, Cx45, Cx50 and Cx57 has been experimentally demonstrated. The lack of a binding site in Cx40h may be due to the absence of two valine residues (arrows) present in Cx40r.

Our data, suggesting that CL2 is most relevant to chemical gating [55,128], agree with evidence of a CaM binding site in Cx43’s CL2 (res. 136–158; Figure 11) [145]. To test the chemical gating efficiency of Cx43 mutants lacking the CaM binding site at CL2, two mutants bound to EYFP (a fluorescent protein) were expressed in HeLa cells. The absence of the site eliminated Ca^2+^-dependent gating, confirming that the CL2 domain (res. 136–158) contains the CaM-binding site relevant to Cx43’s Ca^2+^ gating [145]. The relevance of CL2’s CaM-binding site (Figure 11) was further confirmed with channels made of Cx43 [38], Cx50 [146] or Cx44 [80,147].

A remarkable study [148] used a synthetic peptide matching the CL2’s CaM-binding domain of Cx43 (res. 144–158; Figure 11) for testing, by small angle X-ray scattering, Ca^2+^-induced conformational changes. Upon peptide interaction, CaM adopted a more globular conformation, indicating that CaM interacts with the peptide in a typical “collapsed” conformation [148].

Xu and coworkers [38] studied whole-cell, patch-clamp N2a cells expressing human Cx43 or Cx40. Ionomycin application to N2a cells expressing Cx43 resulted in a threefold increase in [Ca^2+^]_i_ and caused Gj to drop by 95%; in contrast, ionomycin did not significantly affect Gj in N2a cells expressing human–Cx40 channels. The chemical gating incompetence of the human–Cx40 [38] at first seems inconsistent with our evidence for the great chemical gating sensitivity of rat–Cx40 channels expressed in oocytes [117], but is consistent with the Ca^2+^–CaM role in gating. In fact, a computer analysis of CL2’s potential CaM binding sites shows that the absence of two residues (V38 and V43) in rat–Cx40, replaced by G39 and A44 in the human–Cx40 (Figure 11), is the likely reason for the predicted inability of the CL2 domain of human–Cx40 to bind CaM [9]. The Ca^2+^-induced drop in Cx43’s Gj was prevented by pretreatment with CDZ, and was reversed by the addition of 10 mM EGTA to Ca^2+^-free salines [38]. The addition of a peptide matching the Cx43’s CL2 CaM-binding domain (res.136–158) to patch–pipette solutions also prevented gating, while neither a scrambled control peptide nor the Ca^2+^/CaM-dependent kinase II inhibitory peptide (res. 290–309) did so [38]. These data indicate that CL2’s CaM-binding domain plays a key role in Cx43’s channel gating.

We have analyzed this CL2 domain as potential CaM-binding site in 13 mammalian connexins by a computer program developed at the University of Toronto (http://calcium.uhnres.utoronto.ca/ctdb/ctdb/sequence.html; Copyright © 2002 Ikura Lab, Ontario Cancer Institute. All Rights Reserved). Significantly, in all of the connexins tested, but human–Cx40, the CL2 domain displays a potential CaM-binding site (Figure 11). The CaM–CL2 interaction has been confirmed experimentally by Jenny Yang’s team for Cx43 [145], Cx44 [147] and Cx50 [146], and by Katalin Török’s team for Cx32, Cx35, Cx45 and Cx57 [111,112].

### 3.8. CaM Is Anchored to Connexins at Resting [Ca^2+^]_i_

Multiple data suggest that CaM is anchored to connexins. One is evidence that the over-expression of CaMCC, a more Ca^2+^-sensitive CaM mutant, drastically reduces the Vj sensitivity of Cx32 channels [107]. Similarly, the Vj sensitivity of Cx45 channels is greatly reduced by inhibition of CaM expression [106]—note that channels made of Cx45 are very sensitive to Vj, and are unique among connexin channels because they close with Vj preferentially by means of the chemical/slow gate [149]. The behavior of heterotypic mutant/Cx32 channels also suggests that CaM is anchored to connexins even at resting [Ca^2+^]_i_ (~50 nM) because the inhibition of CaM expression in mutant/Cx32 channels drastically reduces the effect of Vj on Gj [105].

Evidence that CaM is anchored to connexins at resting [Ca^2+^]_i_ has recently been confirmed by in vitro experiments testing CaM-binding to peptides matching the CL2’s CaM site of Cx32, Cx35, Cx45 and Cx57, in the presence and absence of Ca^2+^ [111,112] (Table 1). In this study, changes in the fluorescence of the double-labelled FRET-probe and Ca^2+^-sensitive TA–CaM were revealed by fluorescence spectroscopy and stopped-flow fluorimetry [140] at physiological ionic strength (pH 7.5, 20 °C). Ca^2+^-dependent and -independent bindings were monitored and the following kD values were obtained (Table 1).

FRET measurements demonstrated partial compaction (54%–70% quenching with Ca^2+^ and 33%–62% quenching without Ca^2+^) of DA–CaM (DDP–maleimide and AEDANS substituted T34C, T110C-calmodulin). Kinetic data showed a two-step process of rapid interaction followed by isomerization, indicating that CaM is anchored to connexins and becomes totally bound upon stimulation [111,112].

## 4. Calmodulin Role in Hemichannel Gating

The existence of connexin hemichannels was first proven in cultured cells expressing Cx43 by evidence of 5(6)-carboxyfluorescein influx with lowered [Ca^2+^] [150]. Hemichannel permeability proved similar to that of gap junction channels and, similarly, hemichannels were sensitive to octanol and heptanol [150]. We further confirmed the presence of hemichannels by demonstrating that the membrane resistance (Rm) of Novikoff hepatoma cells drastically drops with no-added-Ca^2+^ solutions [150]. In control cells, Rm was lower than in cells transfected with anti-sense Cx43 (~800 and ~4000 MΩ, respectively), proving that the number of open hemichannels in low Ca^2+^ saline is much lower than in controls [150].

While external Ca^2+^ clearly plays a major role in keeping hemichannels closed [151,152], an increase in [Ca^2+^]_i_ actually causes hemichannel opening [103,104,153]. In Cx32-expressing cells, a [Ca^2+^]_i_ rise to ~500 nM, caused by treatment with 2 μM A23187 (a Ca^2+^ ionophore), triggered ATP release and dye uptake that was blocked by a Cx32 mimetic peptide [104]. Significantly, this peptide (“32gap 24”; GHGDPLHLEEVK, res. 110–121) mimics a CL sequence that just precedes the CaM binding site (Figure 11). Hemichannel opening was prevented by W7 [104], suggesting a CaM role in hemichannel gating that is opposite its role in cell-to-cell channels. A subsequent study confirmed these data on Cx43 hemichannels expressed in glioma cells and primary glial cells [103]. Surprisingly, however, while a [Ca^2+^]_i_ rise to ~500 nM opened hemichannels, this phenomenon vanished with a greater [Ca^2+^]_i_ rise. Note, however, that the hemichannel closure at high [Ca^2+^]_i_ is likely to be CaM-independent [154]. CaM’s role in hemichannel gating has been also reported for Cx50 hemichannels expressed in HeLa cells [125].

The gating mechanism of hemichannels and the role of CaM in hemichannel opening and closure are still poorly understood. A recent study [153] reported that a CT-deleted Cx32 mutant (Cx32–D220) renders hemichannels less sensitive to [Ca^2+^]_i_; significantly, Ca^2+^-sensitivity is restored by application of the peptide “32gap 24”. These Authors suggested that the interaction of “32gap 24” with the Cx32–D220 hemichannel stabilizes CL fluctuations, and proposed that CL fluctuations may prevent the exposure of CL residues to a target domain relevant to gating [153]. In agreement with our “CaM–Cork” gating model [9,10,16], they believe that Cx32 hemichannels are kept closed at resting [Ca^2+^]_i_ by a plugging molecule likely to be a CaM lobe [153]. Also consistent with the idea that a CaM lobe plugs the hemichannels (cork gating), is evidence that the hemichannels are opened by positive (depolarizing) voltage pulses [153,155]. Significantly, Castro and coworkers directly measured the opening and closure of Cx32 hemichannels by patch-clamp in response to a [Ca^2+^]_i_ rise, hence the kinetics of the hemichannel’s “cork” unplugging [155]. Indeed, based on the CaM–Cork, model the negatively charged CaM lobe is expected to be displaced out of the positively charged hemichannel’s mouth (vestibule) by membrane depolarization caused by positive voltage pulses [9].

A recent study on a Cx46 mutant (G143R) confirmed the direct CaM role in hemichannel gating [156]. The G143R mutation in the CaM-binding site (Figure 11), which increases hemichannel permeability [157], affected CaM binding to CL2 [156]. As predicted, both CaM binding to Cx46’s G143R mutant and increased hemichannel permeability were inhibited by CDZ. Significantly, G143R substitution greatly increases the CaM–Cx46 interaction in the presence and absence of Ca^2+^ [156], confirming that that CaM is anchored to connexins at normal [Ca^2+^]_i_ [111,112]. Perhaps, the enhanced Ca^2+^–CaM affinity of the G143R mutant is caused by a slight shift in the site toward the COOH-terminus end [9]. The CaM–Cx46 interaction was also confirmed by immunofluorescent CaM–Cx46 co-localization [156].

Recently, Garcia and coworkers reported that the mutation G12R in Cx26’s NT increases the kinetics speed of slow-gate closure and, although the hemichannels still close completely at a very negative Vm, they are not affected by Ca^2+^, even though Ca^2+^ still binds [158]. While it might be irrelevant to this phenomenon, it is noteworthy to realize that the G12R mutation reduces the extent of the NT’s CaM-binding site [9]. Another mutation (N14K) [159] decreased the extent of the CaM binding site even more [9]. The N14K mutation raises the energy barrier between open and closed hemichannel states and shifts calcium sensitivity, voltage sensitivity and deactivation time constants [159].

## 5. Chemical Gate, Slow Vj-Gate and Calmodulin

Gap junction channels are thought to have five types of gate: fast Vj-gate, slow Vj-gate, chemical gate, Vm-sensitive gate and extracellular gate (sensitive to Ca^2+^ in hemichannels). The slow Vj-gate and the Vm-sensitive gate behave like the chemical gate in terms of kinetics and efficiency [160,161]; therefore, we believe that they are the same gate. Consequently, this gate will be here named the “chemical/slow gate”.

**Figure 12:**
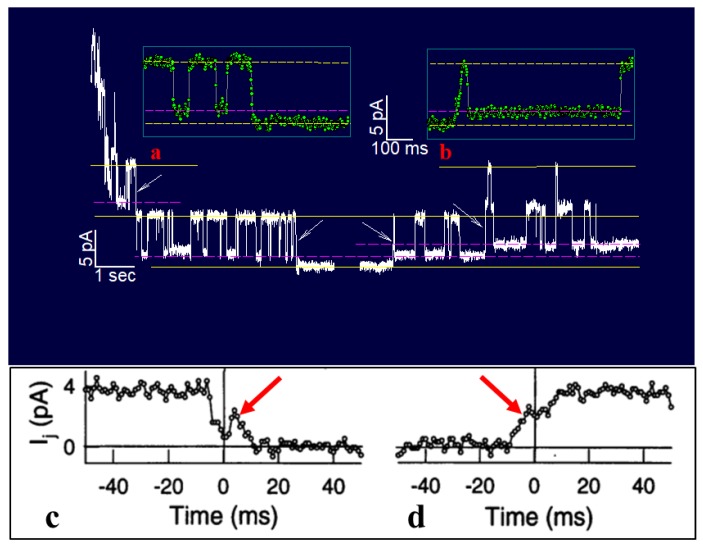
Junctional current (Ij) and single channel conductance (γj) monitored by a double, whole-cell clamp in HeLa cells stably transfected with Cx43, during uncoupling by 100% CO_2_. At Vj = 55 mV, the fast Vj-gating of the last five open channels (left side) is manifested by quick (~2 ms) Ij flickering between open (γj main state; top yellow line), and residual (γj residual; pink dashed-line), states, with a γj (residual)/γj (main state) ratio of 20%–25%. In contrast, the chemical/slow gate closes the channel completely (bottom yellow line) by a slow Ij transition (~10 ms; insets **a** and **c**). Each channel reopens by a slow transition (~10 ms) from closed to open state (right side and insets b and d). Sampling points (green dots in **a** and **b**, and circles in **c** and **d**) were recorded at 1 ms intervals. The slow transitions often display fluctuations during channel closing (inset c) or opening (inset d), suggesting that a large particle, like a CaM lobe, flickers in and out of the channel’s mouth (or competes with other CaM lobes) before settling in its final position. Adapted from Reference [161].

Bukauskas and I have monitored single channel’s gating behavior in rat fibroblasts and HeLa cells transfected with Cx43 during exposure to 100% CO_2_ [161]. Junctional current (Ij), single channel conductance (γj) and Ij-kinetics were recorded during uncoupling and recoupling at different Vj-gradients, in order to distinguish chemical/slow gate and fast Vj-gate behaviors. At Vj = 55 mV, both gates are active: the fast Vj-gate displays fast Ij flickering between open γj (main state) and residual γj (residual) states, while the chemical/slow gate shows slow Ij transitions between open and closed states (Figure 12, left arrows and inset a) [161]. During recoupling, each channel reopens by a slow transition from closed to open state, followed by fast Ij flickering between open and residual state (Figure 12, right arrow and inset b). Significantly, the transitions from open to closed state, and vice versa, often display fluctuations (Figure 12c,d) [161]. Therefore, the CO_2_-induced chemical gating of Cx43 channels exclusively involves the chemical/slow gating mechanism [161]. These data are consistent with earlier findings on insect cells [162] and mammalian cells expressing Cx40 [163].

While in most connexin channels the chemical/slow gates are preferentially in open state, in Cx45 expressing cells, many of them are spontaneously closed at resting [Ca^2+^]_i_ and pH_i_ [106,149]. Similarly, the chemical/slow gate is spontaneously closed in a variety of heterotypic mutant Cx32 and mutant Cx26 channels [105,135].

We have tested several Cx32 mutants and a Cx26 mutant that generate heterotypic channels that are spontaneously closed by the chemical/slow gate at Vj = 0 and are opened by positive Vj-gradients at the mutant side [105,135]. The Cx32 mutants tested were: tandem, 5R/E, 5R/N, ML/NN, ML/CC, ML/EE, 3R/N and ML/NN+3R/N. In tandems, two Cx32 monomers are bound NT-to-CT. In 5R/E and 5R/N, five arginines of CT (R215, R219, R220, R223 and R224) are replaced by glutamates (E) or asparagines (N), respectively. In ML/NN, ML/CC and ML/EE, two CL residues, methionine (M105) and leucine (L106), are replaced by N, cysteines (C) or E, respectively. In 3R/N, R215, R219 and R220 are replaced by N. In ML/NN+3R/N, two previously listed mutations are combined. 

**Figure 13:**
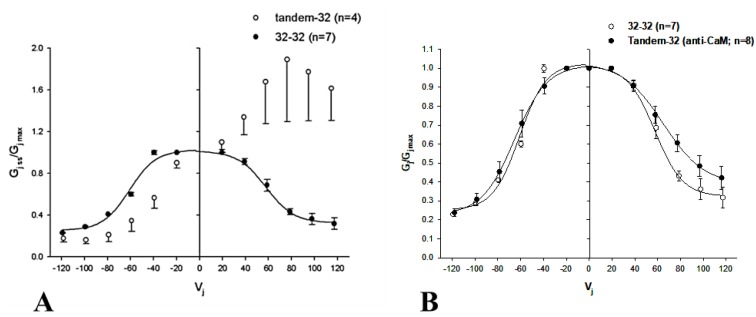
Vj sensitivity of homotypic Cx32 (32–32) or heterotypic tandem–Cx32 (tandem-32) channels monitored in *Xenopus* oocyte pairs. With the application of Vj in steps (20–120 mV), 32–32 channels display a typical Vj sensitivity, as Ij decays exponentially for Vj > ±20–40 mV (**A**,**B**). In contrast, tandem–32 channels display a unique Ij–Vj behavior: with a negative mutant side, initial and final Ij progressively decrease to very low values, while with a positive mutant side, Ij progressively increases to high values. The large asymmetry in the relationship between Vj and normalized Gj (G_jss_/G_jmax_; (**A**) is eliminated by the inhibition of CaM expression (**B**). From Reference [105].

In view of the fact that the chemical/slow gating behavior of these heterotypic channels is qualitatively the same, here we will focus on heterotypic tandem–Cx32wt channels (tandem-32). While homotypic Cx32 junctions (32–32) display a typical Vj sensitivity (Figure 13A), tandem-32 channels show a unique Ij–Vj behavior [135]. With a negative mutant side, as Vj is increased stepwise from −20 to −120 mV, Ij gradually decreases to very low values, and Vj sensitivity is manifested even at the lowest Vj. In contrast, with a positive mutant side, Ij gradually increases to high values, as lots of closed channels become operational. This results in great asymmetry in the relation between Vj and normalized Gj (Gjss/Gjmax) (Figure 13A). This Ij/Vj behavior indicates that Vj-positive or -negative at the mutant side opens or closes, respectively, a greater number of channels [105,135]. Significantly, the Vj-Gjss/Gjmax asymmetry (Figure 13A) vanishes with the inhibition of CaM expression (Figure 13B) [105].

**Figure 14:**
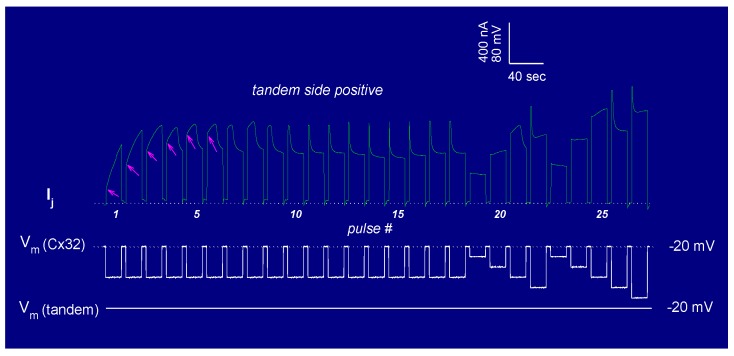
Junctional current (Ij) and Vj sensitivity monitored in *Xenopus* oocyte pairs expressing heterotypic tandem–Cx32 channels. With trains of 60 mV Vj pulses positive at tandem side, three Ij behaviors are observed: monophasic Ij increase (pulses #1–3); biphasic Ij time-course (pulses #4–9) and conventional Ij behavior (pulses #10–18). This suggests that repeated application of Vj pulses, positive at the tandem side, renders all of the available channels (from pulse #10 onwards) progressively operational. Indeed, the application of the conventional Vj protocol to either oocyte immediately after the trains of 60 mV pulses results in a behavior very similar to that of 32–32 channels (pulses #19–27). Adapted from Reference [135].

The tandem-32 channel behavior was also tested by applying trains of long, 60 mV Vj-pulses that were positive at the tandem side (Figure 14) [135]. Three distinct Ij behaviors were seen: a monophasic Ij increase (pulses #1–3), a biphasic Ij time-course (pulses #4–9), characterized by an initial progressive Ij rise followed by exponential decay, and a conventional Ij behavior (pulses #10–18), represented by an initial Ij peak followed by exponential decay to a steady-state level (Figure 14). This indicates that the repeated application of Vj pulses positive at the mutant side gradually opens (renders operational) all of the “dormant” channels, eventually allowing the fast Vj-gate behavior of the adjoined Cx32wt hemichannels to be revealed (Figure 14), after the inhibition of CaM expression (Figure 13B) [105]. In fact, the application of conventional Vj-protocols immediately following the train of 60 mV-positive pulses briefly results in a normal behavior, similar to that of 32–32 channels [135]. The reason for this phenomenon is that Vj pulses positive at the tandem side made most or all of the available channels momentarily operational; thus, with all or most of the chemical/slow gates momentarily in open state, the normal activity of the fast Vj-gates of both tandem and wild-type Cx32 is manifested [135].

The slow change in Gj was interpreted as a gating phenomenon based on the activity of the chemical/slow gate, clearly distinct from that of the fast Vj-gate. There are several reasons for making this distinction [135], one being that, in all of the connexins tested, the chemical/slow gate always closes at the negative side of Vj [105,135], while the fast Vj-gate closes at the negative (Cx32) or positive (Cx26) side of Vj, depending on the type of connexin expressed. Indeed, heterotypic channels between Cx26 and a Cx26 mutant (4pos/E), in which the four basic residues of CT were mutated to E (4pos/E-26), behaved qualitatively as a heterotypic tandem-32, 5R/E-32 and other mutant-32 channels [105,135] (see previously) when exposed to steady-state Vj gradients [9,16] (Table 2).

This is significant because the fast Vj-gates of Cx26 and Cx32 are sensitive to opposite voltage polarities—Cx32 is a “negative gater” while Cx26 is a “positive gater” [164]. This obviously indicates that in both Cx32 and Cx26 mutant channels, this gating behavior is a manifestation of the activity of the negatively charged chemical/slow gate (CaM’s N-lobe, see the following).

One may question why most diverse mutations unmask a similar slow gating behavior. We feel that, while without uncouplers the chemical/slow gate of most connexins, perhaps except Cx45 channels [106,149], is open, certain mutations unbalance the gating state of the mutant hemichannel, favoring the closed state to different degrees. This would allow the negatively charged chemical/slow gate, likely to be a CaM lobe, to gain access to the channel’s mouth (vestibule) and plug it (CaM–Cork gating; see in the following). Closed and open states could be interconverted by Vj, with Vj-positive and -negative at the mutant side, opening and closing, respectively, the mutant hemichannel. 

**Figure 15:**
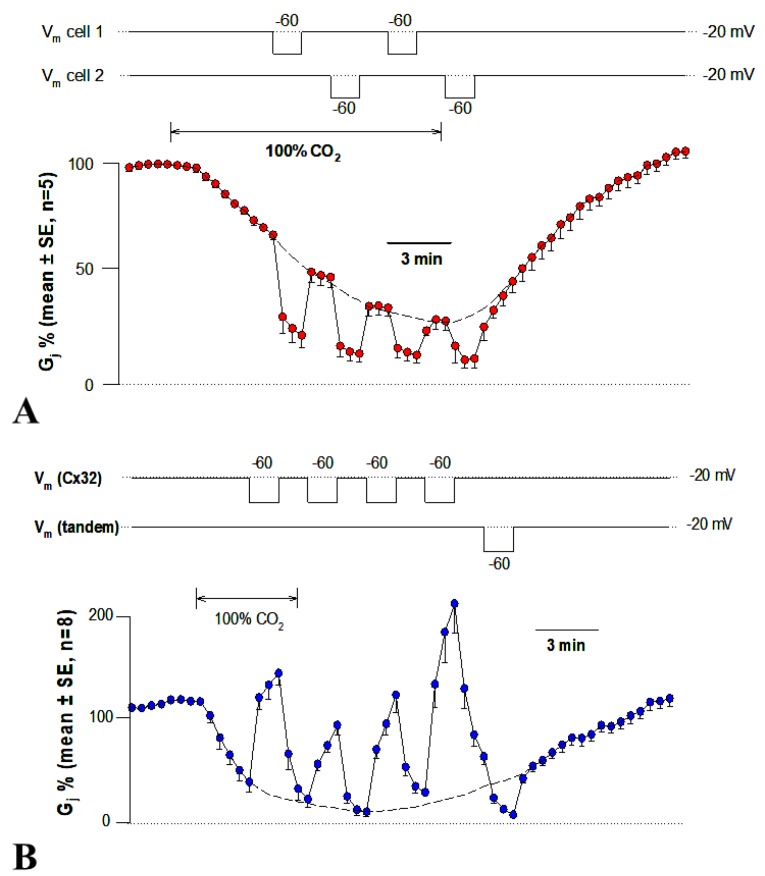
Junctional conductance (Gj) and Vj sensitivity monitored in *Xenopus* oocyte pairs expressing homotypic Cx32 (32–32; **A**) or tandem–Cx32 (tandem-32; **B**) channels, exposed to 100% CO_2_. With 32–32 channels, 40 mV Vj-gradients of either polarity always cause a Gj drop (**A**). In contrast, with tandem–32 channels, Gj increases with 40 mV Vj-gradients positive at the mutant side, and decreases with negative Vj (**B**). The effect of positive Vj progressively decreases as uncoupling progresses (**B**). This confirms evidence that the chemical/slow gate is Vj-sensitive, and suggests that there are two gating states: Vj-reversible and Vj-irreversible. Adapted from Reference [135].

Data generated by experiments in which mutant–Cx32 or Cx32–Cx32 channels were subjected to Vj-gradients of different polarity during CO_2_ exposure (Figure 15) suggest that the chemical/slow gate may close the channels by two different mechanisms [105,135]. With 32–32 channels, Vj gradients of either polarity always resulted in a significant Gj drop (Figure 15A). In contrast, with mutant-32 channels Gj, progressively reduced to lower values by CO_2_ at Vj = 0 mV, dramatically and reversibly increased with Vj gradients positive at the mutant side (Figure 15B) [105,135]. Significantly, with all of the heterotypic mutant-32 channels, the effect of CO_2_ on Gj was minimal following the inhibition of CaM expression [105].

Vj-positive at the mutant side was gradually less efficient in raising Gj as uncoupling developed, and gradually more efficient during recovery (Figure 15B) [107,135]. This suggests that we are dealing with two populations of gated channels: one in *closed state 1* and the other in *closed state 2* [135]. In *closed state 1,* the gates of the mutant hemichannels can be opened by positive Vj, while in *closed state 2* they cannot. We previously named the two gating types *Cx-driven*” and “*CaM-driven*”, respectively [10]. We have now renamed them *CaM-Cork* gating and *Ca–CaM-Cork* gating, respectively [9] (see the following).

**Figure 16:**
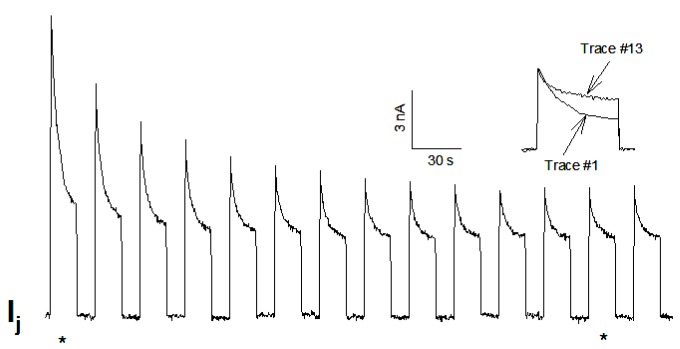
Junctional current (Ij) and Vj sensitivity monitored in *Xenopus* oocyte pairs expressing Cx32 channels. A series of long (12 s, 30 s intervals) 100 mV Vj pulses progressively decrease peak and steady-state Ij by 50%–60% (τ = ~1.2 min). I_jpeak_ drops more dramatically, such that I_jss_/I_jpeak_ increases from 0.4 to 0.6 (see inset). From Reference [115].

While we previously felt that, with most connexins (aside from Cx45), the chemical/slow gate is inactive in the absence of uncouplers or Cx-mutations, our 2007 data indicate that this gate can also be activated by applying a series of large Vj gradients (Figure 16) [115]. With Cx32 channels, the application of a series of −100 mV Vj-pulses caused both peak (Ijpeak) and steady-state (Ijss) Ij to progressively and exponentially drop by 50%–60% (Figure 16) [115]. Gj, measured during recovery by applying small Vj-pulses slowly recovered, often reaching values greater than initial ones. Similar, but even more drastic, results were obtained with the mutant Cx32–D225 [115] that lacks fast Vj-gating [165], as CT-deleted Cx43 [165,166] and Cx40 [167].

These data clearly confirm the idea that the gate responsible for this phenomenon is the chemical/slow gate, rather than the fast Vj-gate. The manifestation of the activity of the chemical/slow gate in wild-type Cx32 indicates that this negatively charged gate, likely to be the CaM’s N-lobe, can be rendered operational even without chemical uncouplers and/or connexin mutations. Indeed, our data [115] confirm previous evidence of sporadic slow-gating events to zero conductance state in cells expressing Cx32 channels subjected to Vj pulses [168].

The phenomena described above are the manifestation of a direct CaM role in chemical/slow gating, because all of them are virtually eliminated by the inhibition of CaM expression [9,105,106]. Furthermore, the inhibition of CaM expression drastically reduced the Vj sensitivity of Cx45’s chemical/slow gate; while, with normal CaM expression, Ij decayed with time for Vj values greater than ±5 mV, following the inhibition of CaM expression, Ij did not decay with Vj values lower than ±40 mV [106]. The inhibition of CaM expression also significantly reduced the CO_2_ sensitivity of Cx45 channels; indeed, with CO_2,_ Gj reversibly decreased by only ~17%, while, in controls, it rapidly dropped to zero (Figure 5B) [106].

## 6. Chemical Gating Model—Direct Calmodulin Role

Gating models not involving CaM have been named: “iris” [169,170,171]; ball-and-chain [57,172,173]; amino–sulfonate [67,71]; *light-switch* [130]; *electrostatic Ca^2+^-mediated* [174] and integrated [175]. Since they are not CaM-related, these models will not be presented here; for their description and discussion, see [9].

### 6.1. The “Cork-Gating” Model

**Figure 17:**
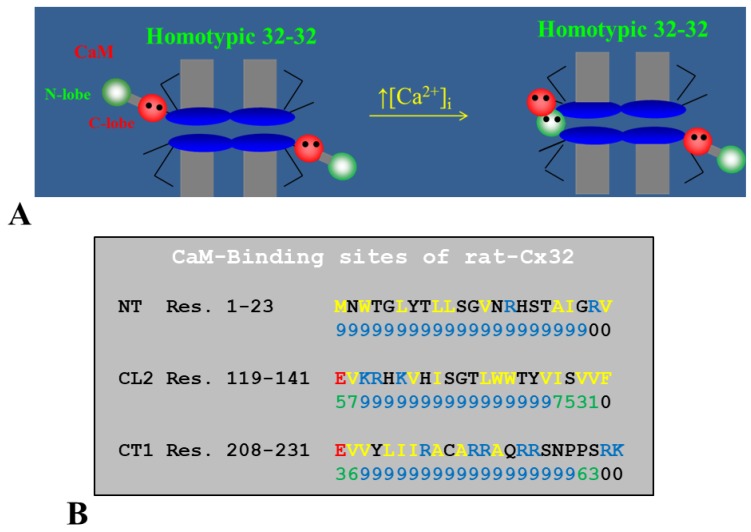
Ca–CaM–Cork gating model (**A**). Gating is believed to involve the physical obstruction of the channel’s by a CaM lobe (**A**). The negatively charged CaM lobe would bind to the positively charged channel’s vestibule hydrophobically and electrostatically, and probably cause conformational changes in connexins as well. Most connexins have three CaM-binding sites (**B**): NH_2_-terminus (NT), the second half of the cytoplasmic loop (CL2), and the initial domain of the COOH-terminus (CT1). These sites (**B**) were proven to bind CaM (see text).

In 2000, we proposed a CaM-based “cork-type” gating model. This model envisions the physical obstruction of the channel’s mouth (vestibule) by a CaM lobe (Figure 17A) [10,16], probably combined with conformational changes in connexins, brought about by Ca^2+^–CaM binding to connexin sites. This model is based on numerous findings suggesting a direct CaM role in chemical gating [9,10,16,80]. As previously mentioned, experimental evidence suggests that the chemical/slow gate is a sizable, negatively charged particle, likely to be a CaM lobe [105,135].

**Figure 18:**
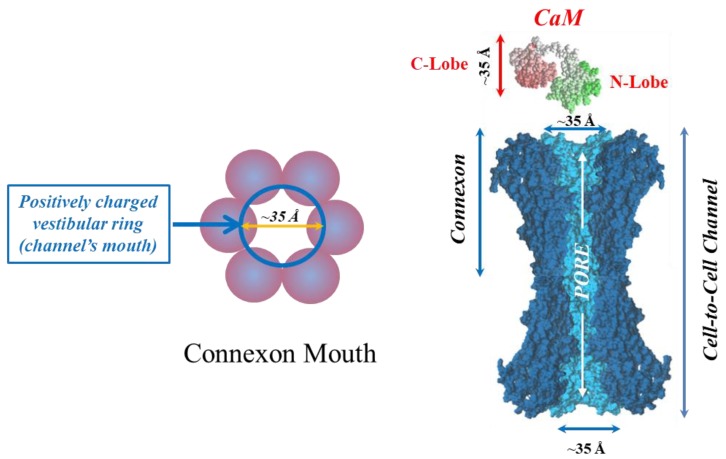
Each negatively charged CaM lobe is ~25 × 35 Å in size (right panel), which is similar to the positively charged channel’s mouth. Thus, a CaM lobe could interact with the connexon’s mouth. In the right panel, the channel is split lengthwise so that the actual pore diameter (light blue area) is visible along the entire channel. CaM and connexons images (right panel) were generously provided by Drs. Francesco Zonta and Mario Bortolozzi (Venetian Institute of Molecular Medicine, VIMM, University of Padua, Italy).

There are many reasons why CaM is the most likely gating candidate. In summary:

1. Chemical gating is sensitive to [Ca^2+^]_i_ in the nM range [4,9,10,80]. Since connexins do not have sequences capable of binding Ca^2+^ in the nM range, gating must be mediated by a CaM-like protein—CaM being the most obvious;
2. CaM binds to connexins [78,79,107,108,127,156];
3. Most connexins have a CaM binding site at NT, CL2 and CT1 domains (Figure 17B) [9,10,80,133]. Most relevant for gating are likely to be the CL2 (Figure 11) and NT sites [9,55,80,111,112,127,128,133];
4. Peptides mimicking the CaM-binding sites of various connexins bind CaM with high affinity [9,55,80,111,112,127,128,133]. Recent data show that in several connexins CaM binds to the CL2 site both in Ca^2+^-dependent and -independent ways [111,112], suggesting that CaM is anchored to connexins at resting [Ca^2+^]_i_;
5. CaM and connexins co-localize at gap junctions and intracellular spots [107,108,114,126,127];
6. Each of the two *negatively* charged CaM’s lobes is ~25 × 35 Å in size [176], which is the same size as the *positively* charged channel’s mouth (vestibule; Figure 18) [177,178,179];
7. Chemical gating is eliminated by the inhibition of CaM expression [73,105,106].
8. CaM blockers (inhibitors) prevent uncoupling by acidification and/or increased [Ca^2+^]_i_ [4,38,46,76,77,93,94,96,97,98,122];
9. Overexpression of CaMCC, a CaM mutant with a higher Ca^2+^-affinity, greatly increases the chemical gating sensitivity of Cx32 channels [107,108];
10. At the single channel level, the chemical/slow gate opens and closes completely and very slowly, and most often displays fluctuations (Figure 12) [161], consistent with the idea that a large particle, likely to be a CaM lobe, flickers in and out of the channel’s mouth before settling in the final position;
11. Channels made of human–Cx40, a connexin that lacks the CL2’s CaM-binding site (Figure 11), are not gated by increased [Ca^2+^]_i_ [38]. In contrast, in channels made of rat-Cx40, which has the CL2’s CaM-binding site (Figure 11), chemical gating is fully functional [117];
12. An increase in [Ca^2+^]_i_ opens Cx32 and Cx43 hemichannels [103,104,153]; this is prevented by W7 [104], suggesting a CaM role in hemichannel gating opposite to that in cell-to-cell channels. The direct CaM role in hemichannel gating was also reported for Cx50 [125] and Cx46 [156] channels.

The cork-gating model envisions two types of CaM-mediated gating: “Ca–CaM–Cork” and “CaM–Cork”. In the former, gating is initiated by Ca^2+^-induced CaM activation. In the latter, gating occurs without a [Ca^2+^]_i_ rise and in most connexins, except in Cx45 [106], would require either a connexin mutation [105,135] or the application of large Vj gradients [115].

#### 6.1.1. Ca–CaM–Cork Gating Mechanism

**Figure 19:**
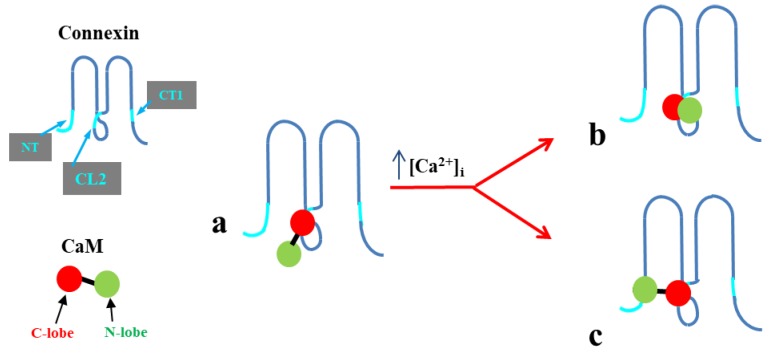
CaM is likely to be anchored to the connexin’s CL2 site by its C-lobe (a). With [Ca^2+^]_i_ > ~50 nM, the N-lobe would gate the channel by binding hydrophobically and electrostatically to the CL2 (**b**) or NT (**c**) domain of the same connexin (trans-domain interaction) or another connexin of the same connexon (trans-subunit interaction).

The Ca–CaM–Cork model proposes that a [Ca^2+^]_i_ rise above resting levels (> ~50 nM) activates CaM and enables a CaM lobe (probably the N-lobe) to plug the channel’s mouth (Figure 17A and Figure 18) [9,10,16]. At resting [Ca^2+^]_i_, CaM is believed to be anchored to each connexin of the connexon by one of its lobes (most likely the C-lobe) at the CL2 site (Figure 19a) [111,112]. The other lobe is likely free, but unable to gain access to the channel’s mouth without CaM’s activation by [Ca^2+^]_i_ higher than resting values. In Cx32 channels, the inaccessibility of the channel mouth may be caused by a postulated CL1–CT1 interaction (Figure 10) [9,74].

The possibility that CaM only binds to connexins when the [Ca^2+^]_i_ increases above basal levels is unlikely because there is evidence for a CaM–connexin co-localization at resting [Ca^2+^]_i_ before and after gap junction formation in cells expressing Cx32 (Figure 7) [107,108], Cx50 [124,125] or Cx36 [114]. This is also consistent with evidence for CaM-dependent gating at resting [Ca^2+^]_i_ in special conditions (CaM–Cork gating; see the following) [9,105,106,115,135]. Evidence that expression of the CaM mutant CaM_1,2,3,4_, which lacks Ca^2+^-binding sites (see previously), prevents the expression of functional gap junctions is also consistent with this idea [10]. In addition, evidence for Ca^2+^-independent CaM-binding to the CL2 site of α, β and γ connexins (Cx32, Cx35, Cx45 and Cx57; Table 1) [111,112] supports the idea that CaM is anchored to connexins at the CL2 site even at resting [Ca^2+^]_i_, and gates with an increase in [Ca^2+^]_i_ above resting values [10,16].

The Ca^2+^-affinity constant of the C-lobe’s EF-hand pair is greater than that of the N-lobe’s pair by almost one order of magnitude (K_d(app)_ = 5.6 and 32 muM for C-lobe and N-lobe, respectively) [109,180]. Therefore, it is likely that the N-lobe interacts with the gating site (CL2 or NT; Figure 19b or c, respectively) only when [Ca^2+^]_i_ increases above resting levels. This model agrees with evidence for the separate functions of CaM’s N- and C-lobes in interacting with Cx32 [133].

**Figure 20:**
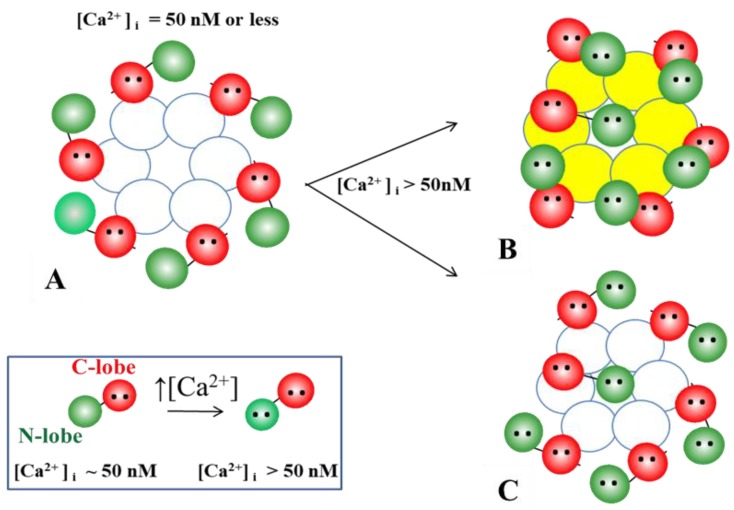
The Ca-CaM-Cork gating model proposes that at normal [Ca^2+^]_i_ (~50 nM) CaM is anchored to each connexin by its C-lobe to the CL2 site (**A**, white-colored connexins). With a [Ca^2+^]_i_ rise, one scenario could be that each N-lobe binds to the NT or CL2 site of the same connexin (*trans-domain interaction*) and change the connexin conformation (B, yellow-colored connexins); this would allow an N-lobe to access the channel’s mouth and plug the pore by binding to the NT or CL2 site of the opposite connexin (**B**, *trans-subunit interaction* – “cork gating”). Another scenario could be that with a [Ca^2+^]_i_ rise all of the N-lobes are activated, but only one binds to a site of the opposite connexin and plugs the pore (**C**, “cork gating”). If this were the case, the first Ca^2+^-activated N-lobe would win the competition (*first come, first served*), preventing other N-lobes from accessing the channel’s mouth.

Although the fine details of the CaM-mediated gating mechanism are still unclear, present data suggest that gating results from the plugging of the connexon’s mouth (vestibule) by a Ca^2+^-activated CaM lobe. The Ca–CaM–Cork gating model proposes that at resting [Ca^2+^]_i_ (~50 nM) CaM is anchored to each connexin by its C-lobe to the CL2 site (Figure 20A, white-colored connexins).

With a [Ca^2+^]_i_ rise, potential scenarios may be as follows:

1. Each of the six CaM’s N-lobes is activated, binds to the NT or CL2 site of the same connexin (*trans-domain interaction*) and changes connexin conformation (Figure 20B, yellow-colored connexins). The connexins’ conformational change allows an N-lobe (negatively charged) to access the channel’s mouth and plug the pore by binding to the NT or CL2 site of the opposite connexin (Figure 20B, *trans-subunit interaction*) and interacting electrostatically and hydrophobically with the positively charged channel’s mouth (“cork gating”; Figure 18);
2. All of the N-lobes are activated, but only one binds to a site (NT or CL2) of the opposite connexin (Figure 20C, *trans-subunit interaction*) and plugs the pore (cork gating) by interacting with the positively charged channel’s mouth electrostatically and hydrophobically (“cork gating”; Figure 18).

In both cases, one N-lobe would win the competition (*first come, first serve*) by plugging the channel’s mouth and preventing other N-lobes from accessing the channel’s mouth.

We have reported that heteromeric 5R/N–Cx32 channels behave as if Cx32 were dominant in determining gating sensitivity to CO_2_, because even one wild-type Cx32 monomer among the six connexins of the 5R/N hemichannel was sufficient to maintain the low sensitivity to CO_2_ typical of wild-type Cx32 channels [129]. Based on our hypothesis that the high CO_2_-sensitivity of the 5R/N-Cx32 channels may result from 5R/N’s inability to establish CL1–CT1 interactions (Figure 10), it would seem possible that this interaction, present just in one Cx32 monomer of each connexon, is enough to partially impair a CaM’s N-lobe’s access to the channel’s mouth [129].

#### 6.1.2. CaM–Cork Gating Mechanism

The CaM–Cork gating mechanism is exemplified by the behavior of heterotypic mutant Cx32 channels [105,135], homotypic Cx45 channels [106] and Cx32 channels subjected to large Vj gradients [115]. The idea is that, at the mutant hemichannel side of heterotypic mutant Cx32 (mutant Cx26) channels, the N-lobe of CaM is able to access the channel’s mouth even at resting [Ca^2+^]_i,_ perhaps because the mutations render the channel’s mouth unprotected. The *negatively* charged CaM’s N-lobe would interact electrostatically with the *positively* charged channel’s mouth, such that it could be displaced by Vj-positive at the mutant hemichannel’s side [9,105,135].

As previously mentioned, the CaM–Cork gating phenomenon unquestionably involves the chemical-slow gate (N-lobe) rather than the fast Vj-gate because the channel is opened by positive Vj at the mutant side both with “negative fast-Vj-gaters” like Cx32 and “positive fast-Vj-gaters” like Cx26 (Table 2) [9,16]. If the fast Vj-gate were involved, Vj positive at the mutant side would close rather than open heterotypic mutant Cx26 channels (4pos/E-26; Table 2).

Cytosolic connexin domains have a high ratio of basic versus acidic residues. For example, in Cx32, if we neglect most of CT, whose deletion by over 80% does not affect chemical gating sensitivity [74], and assign charge values of 1 for R, K, D and E residues and 1/2 for H residues, we find 18 basic and six acidic residues per connexin—108 and 36 residues, respectively, per hemichannel (connexon). Based on the short range of electric field’s effectiveness, the Vj-sensitive slow gating behavior of heterotypic mutant Cx32 channels could only manifest itself if the gating CaM lobe were always very close to the channel’s mouth.

One may question why the channel’s mouth (vestibule) would be freely accessible to the gating CaM’s N-lobe in Cx32 mutants only. Possibly, in wild-type connexins the inaccessibility of the channel’s mouth may result from the postulated CL1–CT1 electrostatic/hydrophobic interaction (Figure 10). The absence of this interaction in mutant channels that lack CT1’s positive charges (5R/N, 5R/T) or in mutant channels in which the charges were converted to negative (5R/E) [74,116,135], and CL1’s hydrophobic residues M105 L106 are mutated to N or E (ML/NN, ML/EE, ML/NN+3R/N) [105], would enable the N-lobe to freely access the channel’s mouth. Similarly, in Cx32-tandem channels the linkage of three NT chains to three CT chains might hinder the postulated CL1–CT1 interaction [105,135].

In view of the effect of large Vj-gradients applied to homotypic Cx32 channels (Figure 19) on Gj [115], it seems likely that the gating particle (CaM’s N-lobe) could be forced to plug the mouth of the hemichannel subjected to negative Vj in the absence of Cx mutations or Ca^2+^-activation. Therefore, the hemichannel’s mouth (vestibule) may not be totally inaccessible, so that the N-lobe could be forced to access it by large negative Vj-gradients (100 mV) (Figure 16) [115]. 

In several wild-type connexin channels, large Vj-gradients may not be needed to force the gating element (CaM’s N-lobe) into the channel’s mouth. Indeed, in cells expressing Cx45, a number of channels are closed by the chemical/slow gate, even at Vj = 0 and without uncouplers [106,149]. Possibly, the postulated CL1-CT1 interaction (Figure 10) is weaker or absent in Cx45 channels. In view of the fact that inhibition of CaM expression eliminates the spontaneous gating of Cx45 channels [106], the gating element is likely to be a CaM lobe.

### 6.2. Locked-Gate Model—Irreversible Channel Gating

In 2012, Xu and coworkers reported that in some cases chemical gating is irreversible [38]. This study tested the Ca^2+^-dependent gating of Cx43 channels in N2a cell pairs exposed to 1 µM ionomycin and 1.8 mM Ca^2+^; competitive peptides or CDZ prevented the Gj drop, confirming the CaM role in gating [38]. To test the uncoupling reversibility, the Ca^2+^-containing solution was switched to a no-Ca^2+^-added solution containing 10 mM EGTA. Significantly, if the switch to no-Ca^2+^-EGTA solutions was made when Gj had dropped by just 50%, Gj recovered almost fully; in contrast, if the solution-switch was done when Gj had dropped to 0% (full uncoupling), Gj recovered to only ~60% [38]. This suggests that there might be two Ca^2+^-induced gating states: one reversible and the other irreversible (locked-gate).

**Figure 21:**
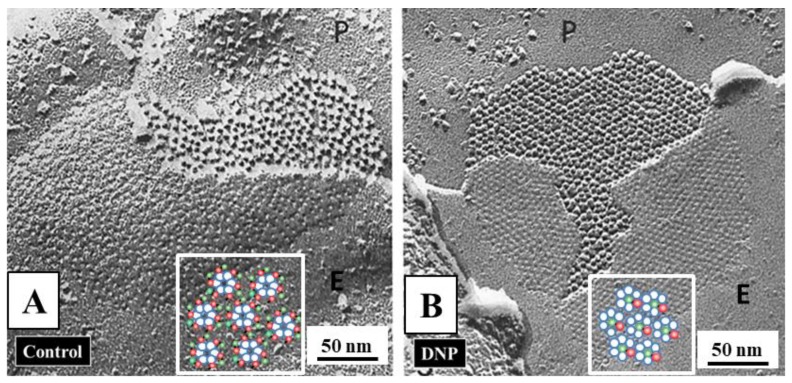
Freeze fracture images of gap junctions from rat stomach epithelium. In controls, most gap junctions display particles (connexons) and pits irregularly packed at center-to-center spacing of 103-105 Å (**A**). In cells uncoupled by 1 h treatment with 2,4-dinitrophenol (DNP) particles and pits aggregate into crystalline, hexagonal, arrays with an average center-to-center spacing of ~85 Å (**B**). We propose that while in coupled conditions CaM is linked to each of the 6 connexins (A, inset), with high [Ca^2+^]_i_ all of the CaM molecules, but the gating one, might detached from connexins, allowing the channels to tightly pack into crystalline (hexagonal) arrays (B, inset). P and E: Protoplasmic and Exoplasmic faces, respectively. Adapted from Ref. [182].

The possibility of a “locked-gate” state [38] brought to mind our early data that demonstrated changes in gap junction channel-packing in freeze-fracture replicas from loose (Figure 21A) to tight-crystalline (Figure 21B) in cells in which [Ca^2+^]_i_ was increased by exposure to uncouplers [2,3,181,182,183,184,185]. We proposed that gap junction crystallization might reflect channel conversion from open to closed state. Based on the observation of Xu and coworkers [38], it is possible that crystallization reflects an irreversible gating state, leading to gap junction internalization, the formation of annular gap junctions, and eventual degradation. Indeed, a study reported the apparent irreversibility of junction crystallization with prolonged exposure to uncouplers, as well as an increased number of annular (internalized) gap junctions [186].

As a hypothesis, we propose that, while in coupled condition CaM is linked to each of the connexins (Figure 21A, inset), with prolonged exposure to high [Ca^2+^]_i_, all of the CaM molecules but the gating one, might become detached from connexins. This would allow the channels to tightly pack into crystalline (hexagonal) arrays (Figure 21B, inset), by interacting hydrophobically with each other. This might explain why isolated, negatively stained gap junctions are crystalline (hexagonal particle arrays), and the channels remain tightly connected to each other even though the junctions are subjected to harsh isolation procedures [2,9,187].

If so, what would cause CaM molecules to be released from connexins at high [Ca^2+^]_i_? In 2004, Black and coworkers developed a genetically encoded fluorescent biosensor to determine the intracellular concentration of both Ca^2+^-free (apo) and Ca^2+^-activated (holo) CaM [188]. This study reported that, in a human kidney cell line stably expressing the biosensor, the [CaM]_i_ at rest is 8.8 ± 2.2 µM. A [Ca^2+^]_i_ rise induced by agonist, store-operated Ca^2+^-entry, or ionophores caused a Ca^2+^-dependent consumption of available CaM, resulting in a [CaM]_i_ drop to ≤200 nM. This is caused by the drastic buffering of free CaM due to excess availability of CaM binding sites. The same results were obtained in cardiac myocytes of adult rabbits. In fact, in both permeabilized and intact myocytes, with a [Ca^2+^]_i_ rise, CaM reversibly moved into the nucleus [189].

The same phenomenon might apply to gap junctions. A potential scenario, therefore, could be that, with a great/prolonged [Ca^2+^]_i_ rise, the five non-gating CaM molecules become detached from connexins and bind to other CaM binding sites with a higher affinity to Ca^2+^–CaM, which would successfully compete against connexins for CaM binding.

## 7. Conclusions

While much still needs to be investigated to understand the gating mechanism of gap junction channels in detail, most relevant, thus far, is evidence that direct cell-to-cell communication is finely modulated by the direct action of Ca^2+^–CaM at nanomolar [Ca^2+^]_i_. This is important not only because this finding brings together the vast knowledge of two major fields of cell biology, but also because it proves that the function of chemical gating is much greater than just protecting healthy cells from damaged or dead neighboring cells (healing over).

Further knowledge of the CaM-role in modulating the permeability of channels and hemichannels made of connexins/innexins may also pave the way for understanding the behavior of intracellular connexin/innexin channels. Indeed, evidence accumulated over several decades indicates that these proteins form hexameric connexons/innexons in non-junctional plasma membranes, the Golgi apparatus, mitochondria and, in some cases, also in the endoplasmic reticulum [9]. Many questions need to be answered, such as: are intracellular connexons/innexons capable forming functional intracellular hemichannels? Do they interact with each other to form “intracellular” junctions? Do they interact with gap junctions? Indeed, some intriguing findings, published over the last four decades and before, have raised the possibility that connexin/innexin-mediated communication might also occur intracellularly between organelles, as well as between organelles and gap junctions, possibly via “inverted” intracellular gap junctions [9].

## Figures and Tables

**Table 1:** Ca-Dependent and -Independent CaM-Binding to CL2 Domains.

Connexins	kD (with Ca^2+^)	kD (without Ca^2+^)
Cx32	40 ± 4 nM	280 ± 10 nM
Cx35	31 ± 2 nM	2.67 ± 0.09 µM
Cx45	75 ± 4 nM	78 ± 1 nM
Cx57	60 ± 6 nM	52 ± 14 nM

**Table 2:** Changes in Gj Caused by Steady-State Vj-Gradients (±40 mV).

Heterotypic Channels	+40 mV at Mutant Side	−40 mV at Mutant Side
Tandem-32	↑262% ± 64%	↓84% ± 11%
5R/E-32	↑182% ± 50%	↓85.2% ± 3.3%
4pos/E-26	↑65% ± 10%	↓35% ± 10%
